# Isoprene‐Emitting Transgenic Tobacco Shapes Root Microbiome and Enhances Growth of Co‐Cultivated Non‐Emitting Plants

**DOI:** 10.1111/pce.70698

**Published:** 2026-07-05

**Authors:** Manuel Bellucci, Mohammad Golam Mostofa, Gian Maria Niccolò Benucci, Ahmad Humayan Kabir, Imran Khan, Marco Lombardi, Vittoria Locato, Gregory Bonito, Francesco Loreto, Thomas D. Sharkey

**Affiliations:** ^1^ Department of Energy Plant Research Laboratory Michigan State University East Lansing Michigan USA; ^2^ Department of Biochemistry and Molecular Biology Michigan State University East Lansing Michigan USA; ^3^ Plant Resilience Institute Michigan State University East Lansing Michigan USA; ^4^ Department of Chemistry State University of New York College of Environmental Science and Forestry Syracuse New York USA; ^5^ Department of Plant, Soil, and Microbial Sciences Michigan State University East Lansing Michigan USA; ^6^ Department of Biology Lamar University Beaumont Texas USA; ^7^ Department of Agroecology Aarhus University Tjele Denmark; ^8^ Department of Science and Technology for Sustainable Development and One Health Università Campus Bio‐Medico di Roma Rome Italy; ^9^ Department of Biology University of Naples Federico II Naples Italy; ^10^ Institute for Sustainable Plant Protection National Research Council of Italy Sesto Fiorentino Italy

**Keywords:** isoprene, metagenomics, microbial communities, neighbour‐plants, rhizosphere, VOCs

## Abstract

Isoprene is the most abundant biogenic volatile organic compound emitted by terrestrial vegetation. Here we report the impact of isoprene on root‐associated microbiomes. Using isoprene‐emitting (IE) transgenic tobacco and isogenic non‐emitting (NE) controls, we performed co‐cultivation experiments in natural soil and analysed plant phenotypes and growth alongside bacterial and fungal communities across root, rhizosphere, and soil niches. NE plants co‐cultivated with IE neighbours displayed increased shoot and root biomass, suggesting interactive belowground functions of isoprene. Amplicon sequencing revealed more growth‐promoting microbiota in root and rhizosphere of IE plants than NE plants. Both bacterial and fungal growth‐promoting microbiota were enriched in IE and NE plants grown in the same pot. However, isoprene‐fumigated plant‐free soils did not replicate these shifts, indicating that plant–microbe interactions are required for the modulation of the soil microbiome. Our results suggest that isoprene acts as a belowground cue influencing microbiome assembly and indirectly enhancing growth in neighbouring plants. This work uncovers a potential ecological role for isoprene, highlighting how plant‐derived isoprene can mediate plant–plant–microbiome interactions and contribute to community‐level processes in the rhizosphere.

## Summary statement

Isoprene emitting tobacco plants affected the soil microbiome, increasing growth promoting bacteria and fungi. When grown together with non‐emitting plants, emitting plants stimulated growth of non‐emitting plants when grown in the same pot.

## 1 Introduction

Plants continuously interact with neighbouring organisms, including other plant species. These interactions are frequently mediated by biogenic volatile organic compounds (BVOCs), which serve as important chemical signals and cues (Ninkovic et al. 2019; Zuo et al. 2025). While the effects of constitutive and stress‐induced BVOCs on aboveground plant–plant interactions have been extensively investigated (Ninkovic et al. 2019; Sharkey et al. 2026), the roles of BVOCs in belowground interactions remain comparatively understudied (Peñuelas et al. 2014). Nonetheless, emerging evidence highlights the significance of BVOCs in shaping root‐associated microbial communities (Tang et al. 2019; Duc et al. 2022).

Roots physically anchor plants to their substrates and allow for water and nutrient uptake. In addition, they serve as a key interface for interactions with microorganisms, including both mutualistic mycorrhizal fungi and nitrogen‐fixing bacteria, as well as harmful pathogens. Within the complex rhizosphere environment, microbes associated with roots and soils emit a plethora of chemically diverse BVOCs into the rhizosphere (Peñuelas et al. 2014; Schenkel et al. 2015; Delory et al. 2016; Massalha et al. 2017; Russo et al. 2022). Root‐derived BVOCs have emerged as important mediators of plant‐microbiome interactions (Sharifi and Ryu 2018; Koprivova and Kopriva 2022), often exhibiting anti‐pathogen activity and contributing to belowground plant defence mechanisms (Schenkel et al. 2015). Moreover, these BVOCs may also function as belowground signal molecules that help recruit beneficial microbial partners to support plant health and stress resilience (Sharifi et al. 2022). Notably, sesquiterpenes such as caryophyllene, released from plant roots, have been shown to affect the composition of root‐associated bacterial and fungal populations, thereby indirectly modulating growth and performance of neighbouring plants (Delory et al. 2016; Kudjordjie et al. 2019).

Isoprene is the most abundant BVOC released into the atmosphere (Guenther et al. 2006; Bellucci et al. 2023; Sharkey et al. 2026), and it is constitutively emitted at easily measured levels by the foliage of about 20% of tree species worldwide (Loreto and Fineschi 2015). Isoprene was initially believed to act as a membrane strengthener or an antioxidant, enhancing plant tolerance to abiotic stresses (Loreto and Schnitzler 2010). However, recent studies suggest a more complex regulatory function, indicating that isoprene modulates the expression of genes and the accumulation of metabolites involved in plant growth and defence pathways (Zuo et al. 2019; Monson et al. 2021; Dani and Loreto 2022; Dani et al. 2022).

While biosynthesis and emission of isoprene by plants is now well‐known (Sharkey et al. 2026), the mechanisms underlying isoprene uptake by plant or microbial cells are not fully understood. Due to its small size, hydrophobic nature, and high volatility, isoprene is generally believed to cross biological membranes via passive diffusion. Supporting this idea, Kessler et al. (2023) proposed that membrane structures involved in VOC emission may also facilitate their uptake without dedicated transporters, consistent with the lack of identified isoprene transporters in bacteria or fungi. Likewise, specific cellular receptors for isoprene in plants or microbes have not been described so far. However, recent studies have shown that microbial exposure to isoprene triggers significant changes in gene expression of the same microbes, suggesting indirect sensing mechanisms, possibly through membrane perturbation or metabolic stress (Crombie et al. 2018; Carrión et al. 2020). Recent studies have revealed a pronounced shift from aboveground to belowground biomass allocation in isoprene‐emitting (IE) plants (Bellucci et al. 2024), but the physiological and ecological significance of this phenomenon remains unresolved. Unlike many other BVOCs, isoprene does not seem to play any direct role in plant communication with other organisms. Early investigations produced inconclusive results (Brilli et al. 2007; Laothawornkitkul et al. 2008), and no concrete evidence has been reported so far to support the role for isoprene in plant‐plant communication. Receptors of plant VOCs are unknown but a few putative plant VOC transporters (odorant binding proteins—OBPs) have been identified (Loreto and D'Auria 2022). However, unlike several monoterpenes produced through the same pathway, isoprene shows negligible affinity for putative plant OBPs, as indicated by molecular docking and molecular dynamics analyses (Giordano et al. 2021).

Prompted by our recent findings demonstrating enhanced root development in IE transgenic Arabidopsis plants (Bellucci et al. 2024), we hypothesised that isoprene may exert additional beneficial effects belowground, potentially extending to neighbouring plants. To explore this possibility, we conducted co‐cultivation experiments using both IE transgenic and non‐emitting (NE) empty vector plants of tobacco (*Nicotiana tabacum*) and Arabidopsis, growing either individually or in combination in the same pot. The objectives of this study were to determine whether isoprene alters the growth and phenotype of neighbouring NE (receiver) plants and, in this case, to investigate whether these effects could be attributed to isoprene‐mediated modulation of root‐associated microbial communities, including both fungal and bacterial taxa.

## 2 Materials and Methods

### 2.1 Plant Materials and Growth Conditions

Seeds of tobacco (*Nicotiana tabacum* cv.‘Samsun’) transformed with isoprene synthase (*IspS*) gene from *Populus alba* to produce IE homozygous plants and the corresponding NE azygous (null segregants empty vector) plants were obtained from Claudia Vickers, University of Queensland. The NE and IE lines used in this study correspond to line 32 produced the highest amount of isoprene compared with the other IE transformed‐tobacco lines (Vickers et al. 2009). More information regarding plasmid construction, transformation of tobacco, and line selection is described in Vickers et al. (2009). IE and NE plants were grown for seed at the same time in separate but matched greenhouses. We have bulked seeds several times and have not seen a decline in isoprene emission rate. We used NE and IE seeds that were grown at the same time to avoid maternal effects. Seeds of IE and NE plants were germinated in trays filled with sterile soil (Suremix growing medium, Michigan Grower Products, MI, USA) and subsequently grown in a greenhouse under a 16 h photoperiod, mean daily light integral of 12 mol m^−2^ d^−1^, and day/night temperature set points of 27°C/22°C for 7 days. IE and NE seedlings were then transferred to 15 L pots filled with homogenised field soil “Yard Blend” from Renewed Earth Incorporated, Kalamazoo, Mich and was 86% sand, 6% silt, 8% clay (loamy sand), pH 7.3. Plants were cultivated under the abovementioned greenhouse conditions. Each pot contained two plants. To test the plant–plant and plant‐soil microbiome interactions, IE and NE tobacco plants were grown non‐mixed (two plants of IE or NE) or mixed (one IE plant + one NE plant, MIX) in the same pot for 6 weeks. For each genotype and condition, 10 independent plants were analysed. Plants were watered with half‐strength Hoagland solution every alternate day. Pots were arranged on greenhouse benches in alternating positions so that all plants were exposed to the same level of isoprene in the air. Importantly, pots containing only NE plants were cultivated in an adjacent but separate greenhouse compartment without the presence of IE plants, preventing exposure to airborne isoprene from IE plants. Three independent replicates were carried out with similar results. Data from the final replicate is shown.

To test direct isoprene effects on root growth, in absence of microorganism, a sterile co‐cultivation assay was performed, using our previously developed Arabidopsis *IspS*‐transgenic lines (B2 and C4) (Zuo et al. 2019; Bellucci et al. 2024). Seeds of B2 and C4 transgenic lines as IE, together with EV‐B3 (referred as EV hereafter) were surface sterilised with 70% ethanol and 5% bleach solution for 5 and 10 min, respectively, followed by washing with sterilised Milli‐Q water five times. Sterilised seeds were placed on germination medium (GM) plates containing ½ Murashige and Skoog (MS) solid medium (0.8% agar and 1% sucrose), supplemented with Gamborg's vitamins solution (Sigma‐Aldrich, Germany). After stratification for 3 days at 4°C in the dark, the plates were vertically placed in the growth chamber under a 16 h:8 h, light:dark photoperiod, 120 μmol m^−2^ s^−1^ photosynthetically active radiation (PAR), 23°C/20°C day/night temperatures, and 60% relative humidity. Two growth conditions were setup: (i) non‐mixed, where EV or IE seedlings were grown individually on the same plate, and (ii) mixed, where EV and IE seedlings were co‐cultivated on the same plate to allow potential inter‐plant interactions. Each plate contained 12 seeds, and the experiment was repeated three times independently to ensure reproducibility.

### 2.2 Soil Treatments With Isoprene

Plant‐free pots containing soil were fumigated with isoprene using a certified 3‐ppm tank (Airgas Inc., Radnor, PA, USA) at a flow rate sufficient to cause significant soil mixing. Each pot was fumigated consecutively for 3 min once per week for 6 weeks. After fumigation, each pot was sealed with a black plastic bag to minimise isoprene release from the soil.

### 2.3 Evaluation of Aboveground and Belowground Plant Traits

Six‐week‐old IE and NE tobacco plants were used for aboveground and belowground phenotypic characterisation. For measuring the leaf area, the 4th leaf from the bottom of the plant was cut and photographed. Leaf area was measured from the photographs using the *Fiji* software (Schindelin et al. 2012). Shoot height was manually determined using a ruler. Shoot fresh weight (FW) was measured using a digital balance. The shoots were placed in paper bags and placed in an oven for 4 days at 65°C to determine the dry weight (DW). Root FW and DW were determined as described for shoot biomass measurement. When IE and NE plants were grown together in the same pot, phenotypic measurements were taken separately for each genotype. Thus, phenotypic measurements were carried out in: (1) two IE plants in one pot (IE); (2) two NE plants in one pot (NE); (3) one IE in a pot also having one NE plant (IE_MIX); and (4) one NE plant in a pot also having an IE plant (NE_MIX). This distinction allowed us to assess genotype‐specific responses within the MIX setup.

Additionally, primary root length in Arabidopsis plants grown under sterile co‐cultivation assay was measured from the photographs taken 6‐day‐post‐germination using *Fiji* (Schindelin et al. 2012). Twenty‐four seedlings per genotype and treatment were evaluated to accurately measure the length of roots from both mixed and non‐mixed setup. The measurements were repeated in three independent experiments for consistency, and data from a representative experiment are shown.

### 2.4 Visualisation of Root Traits Using Rhizotron

After germination, 7‐day‐old plants were transferred and grown for further 4 weeks in a transparent rhizotron placed at an angle of 45 degrees with the transparent side facing downward to encourage roots to grow along the visible surface. The transparent side was covered with a black cloth except when observations were being made. Roots of IE and NE plants were separated from soil, washed with water, and photographed with a Fujifilm X‐T2 digital camera and root traits were analysed with RhizoVision explorer (Seethepalli et al. 2020).

### 2.5 Measurement of Isoprene Emission From Roots and Soil

The samples from rhizotron experiment were used to determine the isoprene synthesis capacity of roots. Five‐week‐old roots of IE and NE plants were harvested, carefully excised and washed with distilled water. Bulk soil samples, collected from the same pots but away from the roots, were also harvested to determine background isoprene levels. Approximately 100 mg of fresh tissue per sample was collected in 5 mL glass vials. and analysed as previously described (Bellucci al. 2024). Particularly, roots and soil were incubated for 4 h at 32°C under a PAR of 300 μmol m^−2^ s^−1^. Isoprene emission from the headspace was measured using a Fast Isoprene Sensor (FIS, Hills Scientific, Boulder, CO, USA). A 3 mL aliquot of headspace gas was injected into the instrument at a flow rate of 400 mL min^−1^ using a gastight syringe. The FIS was calibrated with a 3.225 ppm isoprene standard (Airgas USA LLC, TX, USA). Isoprene emission rates were calculated per gram of root/soil FW. For each genotype, six plants were used.

### 2.6 Sample Collections for Microbiome Analysis

Bulk soil and roots with rhizospheric soil (Supporting Information S1: Figure S2) were collected separately in 50 mL Falcon tubes according to a published protocol (Simmons et al. 2018), with few modifications. Bulk soil was collected with a sterile soil core collector from beneath the pots without touching the roots. An epiphyte removal buffer was added to the 50 mL Falcon tubes containing roots and rhizosphere soils to separate the rhizosphere soils from the roots. Falcon tubes were then sonicated at 4°C with 1‐min‐long 160 W pulses, separated by 1‐min‐long rests for 5 min, and further centrifuged at 5000 *g* for 10 min. After separation from the rhizosphere, roots were washed three times and stored at −80°C.

### 2.7 DNA Extraction, Amplicon Library Preparation, and Sequencing

The DNA extraction from roots, bulk soils, and rhizosphere soils was performed with DNeasy Plant Pro mini and DNeasy Power Soil pro kits (Qiagen, Hilden, Germany). The 16S rRNA gene's V4 hypervariable region and the internal transcribed spacer 1 (ITS1) were amplified with bacterial 515f/806r (Caporaso et al. 2011), and ITS1F/ITS2 (White et al. 1990; Gardes and Bruns 1993) primers, respectively. Illumina libraries were prepared and sequenced by the RTSF Genomic Core at Michigan State University using MiSeq v2 250PE chemistry and Illumina‐compatible primers following the protocol described in (Kozich et al. 2013) and mentioned in detail in the Supporting Information S1: File S1.

### 2.8 Metagenomics Analysis

Fungal ITS and bacterial 16S rDNA sequenced libraries were barcode‐based demultiplexed with a custom‐developed pipeline (https://github.com/Gian77/Demultiplex-USEARCH) based on USEARCH (Edgar 2010). Demultiplexed fastq samples were further analysed using the Cecilia pipeline (https://github.com/Gian77/Cecilia). Briefly, the Cecilia pipeline uses FastQC (Andrews 2010) to assess sequencing quality, Cutadapt (Martin 2011) to remove Illumina adapters and sequencing primers, USEARCH to quality filtering, and trimming, de‐replicating and removing singletons, and clustering sequences into operational taxonomic units (OTUs) at 97% similarity using UPARSE algorithms (Edgar 2013; Edgar and Flyvbjerg 2015). Cecilia also implements CONSTAX2 (Liber et al. 2021) to assign taxonomic names to OTU representative sequences with the UNITE eukaryote database (ver. 9, 07.18.2023; Abarenkov et al. 2024) for ITS and SILVA (version 138) (Quast et al. 2012) for 16S.

### 2.9 Statistical and Bioinformatics Analysis

Microbiome (fungal and bacterial OTU tables, metadata, taxonomy, and sequence data) and physiological data were imported into R (R Core Team, 2024). Differences in root growth between IE and NE were assessed using Welch's test (*p* ≤ 0.05). Differences in plant growth, between IE, IE_MIX, NE, and NE_MIX, were assessed using a non‐parametric Kruskal‐Wallis (and ANOVA) test followed by Wilcoxon rank‐sum tests (and Tukey's test) with Benjamini & Hochberg correction (*p* ≤ 0.05). Parametric and non‐parametric tests gave identical results. Microbiome datasets were grouped into a phyloseq object for data mining, subsetting, and exploration convenience with the *phyloseq* package (McMurdie and Holmes 2013). OTUs not classified as fungi or bacteria were removed. One contaminant OTU was identified in the fungal dataset using the *decontam* package (Davis et al. 2018) and removed accordingly. Distributions of sample sequencing depth (Supporting Information S1: Figure S3a) and richness (Supporting Information S1: Figure S3b), as well as rarefaction curves, were generated for both fungi (Supporting Information S1: Figure S4a) and bacteria (Supporting Information S1: Figure S4b), revealing lower depth and richness in root samples compared to rhizosphere and soil for the bacterial community. For this reason, data from each niche was rarefied independently to a consistent sequencing depth. For bacteria, rhizosphere and soil samples were rarefied at 31 244 minimum sequences per sample, while root samples were rarefied at 440 minimum sequences per sample (Supporting Information S1: Figure S5). For the fungi, root, rhizosphere and soil samples were rarefied at 19 849 minimum sequences per sample (Supporting Information S1: Figure S6). While varying rarefaction depths were applied to each niche in the dataset, direct comparisons across niches are limited; however, such comparisons were not within the intended scope of this study. Our primary objective focused on the individual analysis of each niche, ensuring a thorough and accurate evaluation of the unique microbial communities present in each environment. The rarefaction process involved resampling the dataset 100 times at the sample depth and calculating the average value using a custom function based on the *vegan* R package (Oksanen et al. 2024).

Beta diversity was analysed by Principal Coordinate Analysis (PCoA) with Bray‐Curtis community distance in *phyloseq*. To assess differences between treatments, we used a permutational multivariate analysis of variance using distance matrices (PERMANOVA) followed by a multivariate homogeneity of groups dispersions (i.e., homoscedasticity) implemented in the “adonis2” and “betadisper” functions in *vegan*. Pairwise “adonis2” and “betadisper” were run to assess differences between treatment levels following the Benjamini & Hochberg *p*‐value correction method for multiple comparisons.

Alpha diversity was analysed using Hill's numbers (Hill 1973) within the *vegan* R package: Hill 0, for OTU richness, and Hill 1, for OTU Shannon diversity. Significant differences between treatment group levels, within each niche of fungi and bacteria microbiome, were conducted using Wilcoxon tests and the Benjamini & Hochberg *p*‐value correction method for multiple comparisons. To identify differentially abundant taxa between treatment levels for each niche, we used pairwise Wilcoxon tests with Benjamini‐Hochberg *p*‐value correction (*p* ≤ 0.05). For each taxon, we used the lowest taxonomic classified rank in the taxonomy table to group all OTUs present in that rank for. This allowed us to have a higher number of observations (OTUs that were classified in that group) for cross‐level comparisons, providing more power to detect significant differences.

All statistical analyses and graphics were run in R (R Core Team, 2025) and plots were generated using ggplot2 (Wickham 2016). Minor label adjustments on figures have been performed using Inkscape (Inkscape Project 2020).

## 3 Results

### 3.1 Root System Architecture and Biomass Accumulation

Representative images of root systems grown in rhizotron and extracted for analysis revealed marked differences between the IE and NE plants when grown in isolation (Figure 1A). Roots from IE plants had a higher density and greater root area compared to NE plants. Quantitative analysis showed that IE plants produced significantly higher (*p* ≤ 0.05) fresh root biomass than that of the NE plants (Figure 1B). Similarly, root dry biomass was significantly greater (*p* ≤ 0.05) in IE plants (Figure 1C). Measurements of root system architecture revealed that total root length was substantially higher (*p* ≤ 0.05) in IE plants compared to NE plants (Figure 1D), and IE plants also exhibited significantly deeper (*p* ≤ 0.05) root growth (Figure 1E). Importantly, isoprene emission measurements showed that IE roots released 0.33 ± 0.06 pmol g^−1^ s^−1^, whereas NE roots exhibited minimal emission, possibly non‐enzymatic (0.029 ± 0.01 pmol g^−1^ s^−1^) (Supporting Information S1: Figure S7). Isoprene in bulk soil samples was consistently below the isoprene detection limit (Supporting Information S1: Figure S7).

**Figure 1:**
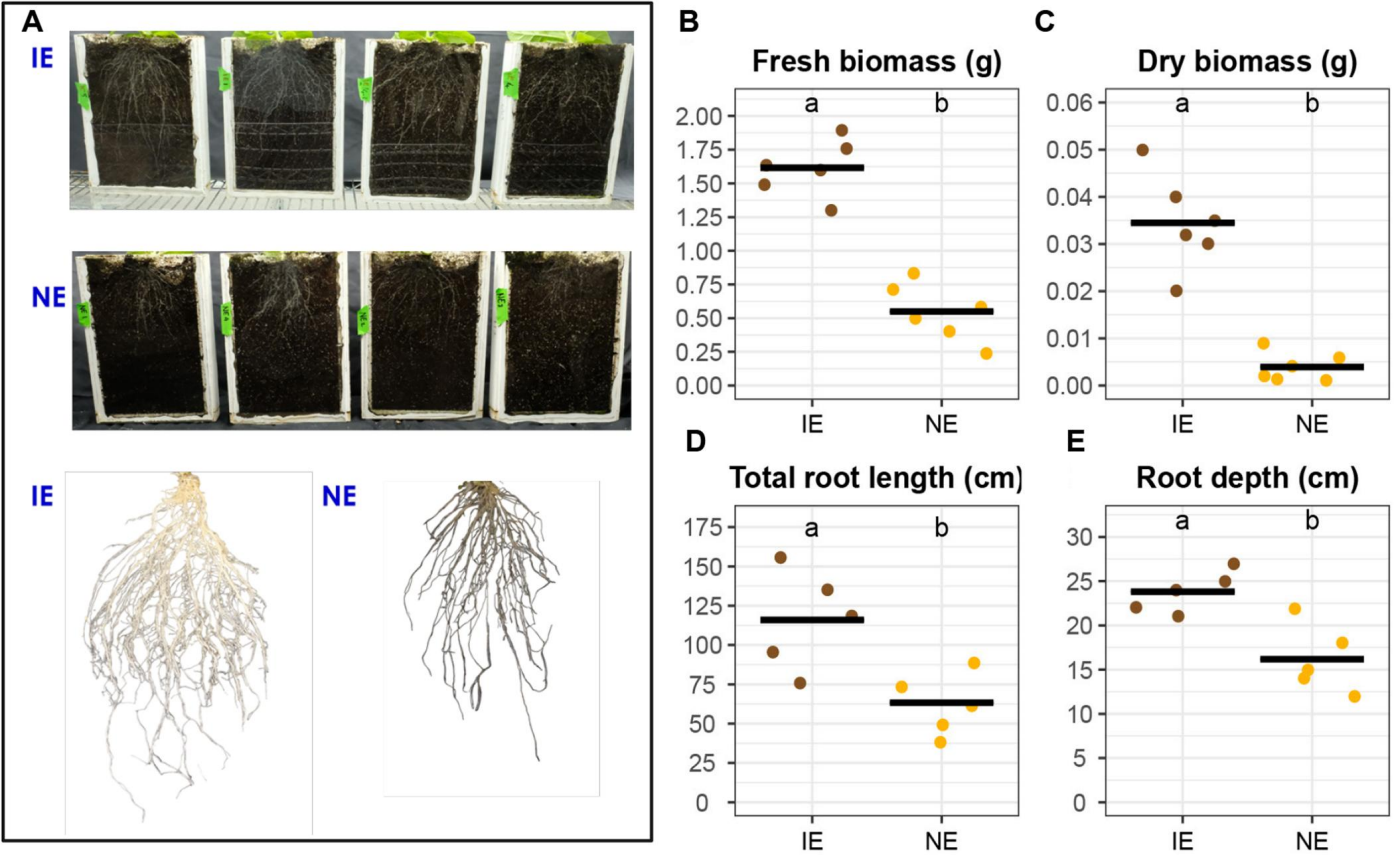
Root growth differences between isoprene‐emitting (IE) and non‐emitting (NE) tobacco plants. (A) Representative images of root systems grown in rhizoboxes (containing only individual NE or IE plants) and after soil removal. (B–E) Quantitative measurements: root fresh biomass (B), root dry biomass (C), total root length (D), and root depth (E). Each point represents an individual plant; horizontal bars indicate mean values (*n* = 5–6). Different letters denote statistically significant differences (*p* < 0.05) between IE and NE plants.

Similarly, transgenic IE Arabidopsis lines (B2 and C4) grown individually under sterile conditions exhibited longer primary root growth compared with EV plants (Supporting Information S1: Figure S8). Importantly, when EV and IE seedlings were grown together in mixed conditions (IE + EV), both genotypes showed a significant increase in primary root length, with EV plants responding to the presence of IE neighbours and IE plants also exhibiting enhanced growth.

### 3.2 Above‐Ground Traits Vary

Visual comparisons of whole plants and leaves revealed marked phenotypic differences among NE, IE, and mixed (IE + NE) plants (see Materials and methods) (Figure 2A–C). IE tobacco plants were typically smaller than NE plants (Figure 2A,B; Zuo et al. 2019). However, in the MIX treatment, both NE and IE plants appeared higher and more robust than the respective plants grown individually in IE and NE treatments. Quantitative measures also supported these observations. Leaf area was significantly greater in NE_MIX and IE_MIX plants compared to that of separately grown IE and NE plants (Figure 2D). Plant height followed a similar trend, with NE_MIX and IE_MIX plants being significantly taller than IE and NE plants, respectively (Figure 2E). Additionally, shoot dry mass of both IE and NE plants significantly improved when they were cultivated together (Figure 2F).

**Figure 2:**
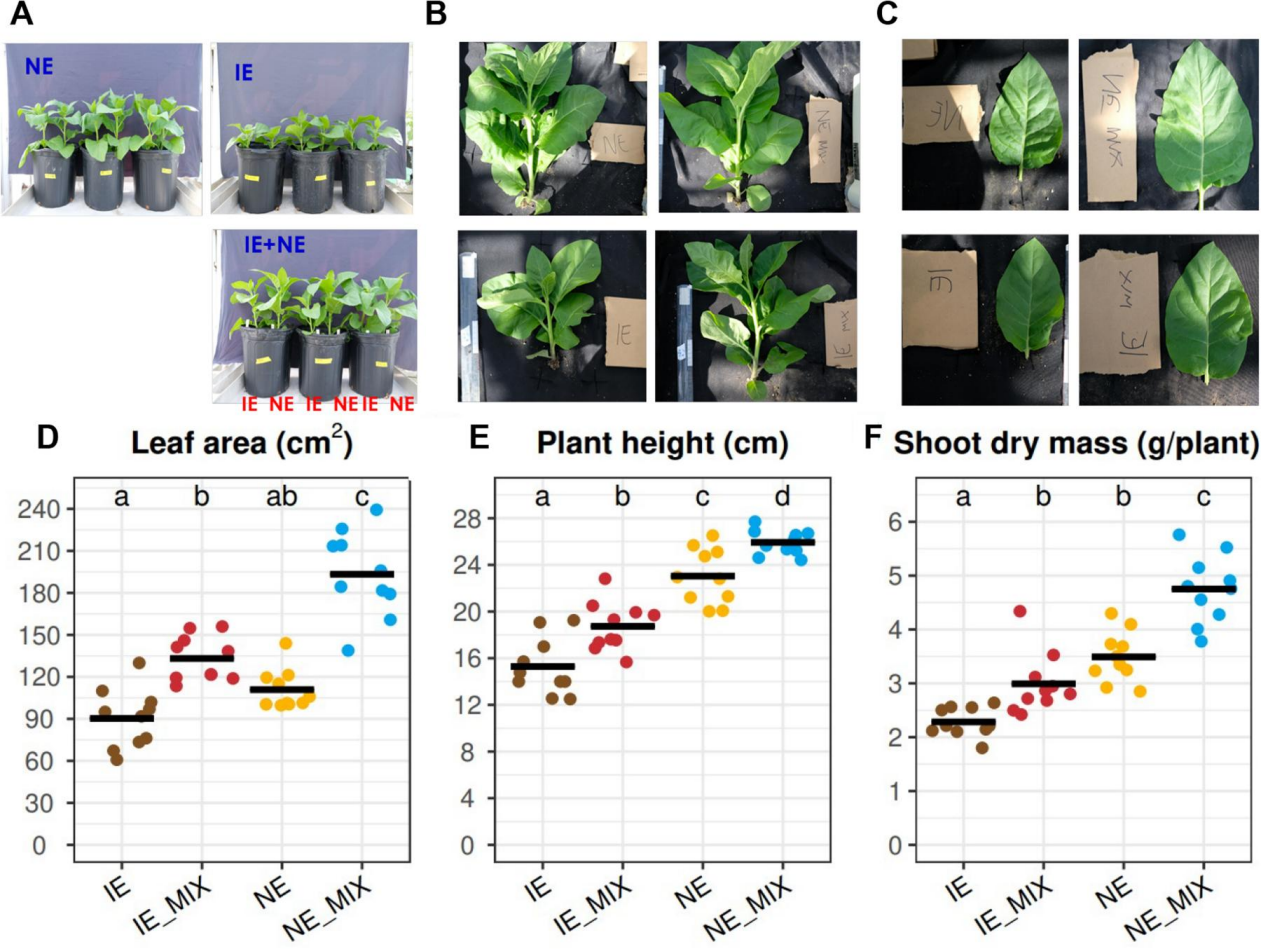
Plant growth in response to co‐cultivation of isoprene‐emitting (IE) and non‐emitting (NE) tobacco plants. (A–C) Representative images of plants grown individually (IE, NE) or together (IE + NE), showing whole plants (A), individual plants (B), and detached leaves (C). Leaf area (D), plant height (E), and shoot dry mass (F) of both types of tobacco plants grown in separate and same pots. Each dot represents an individual plant; horizontal bars show means (*n* = 10 plants). Different letters denote statistically significant differences (*p* < 0.05) among the tobacco plants. The images labelled as IE + NE (Figure 2) correspond to the samples represented in the graphs as IE_MIX and NE_MIX. These indicate co‐cultivation of emitter and non‐emitter genotypes. [Color figure can be viewed at wileyonlinelibrary.com]

### 3.3 Microbial Amplicon Sequencing Results

Illumina sequencing of the fungal and bacterial amplicon libraries resulted in 7 636 598 and 7 595 420 250 ‐bp paired‐end sequences after demultiplexing. After the bioinformatics analysis, a total of 5 137 865 (56 460.1 ± 20 827.9) fungal and 3 982 742 (43 766.4 ± 31 717.5) bacterial, 225 bp long; sequences were retained. Community analysis was run on rarefied data, which accounted for 2396 OTUs, with 19 849 sequences per sample (1 786 410 total sequences) in the fungal dataset and 15 314 OTUs with 43 766.4 (3 982 742 total sequences) in the bacterial dataset, across 92 samples.

### 3.4 Beta Diversity

Fungal and bacterial PCoA showed variable levels of clustering of samples around treatment levels for root, rhizosphere, bulk soil, and control soil in both fungi (Figure 3A–D) and bacteria (Figure 3E–H). The PERMANOVA analysis (Table 1) showed that the most [*R* ^2^ significant (*p* ≤ 0.05)] effect of treatment was the rhizosphere, for both fungi [*R* ^2^ = 22.3%, *F*_(3,2.294)_] and bacteria [*R* ^2^ = 27.1%, *F*_(3,2.969)_]. Beside the rhizosphere, only root [*R* ^2^ = 23.5%, *F*_(3,2.359)_] and the control soil [*R* ^2^ = 17.3%, *F*_(1,2.509)_] niches showed significant (*p* ≤ 0.05) differences between treatment levels, with the root niche having significant dispersion [*F*_(3,3.643)_] between sample groups (Table 1). We did not detect any difference between soil samples, either in the fungal or bacterial datasets, as these communities are less dependent on the plant.

**Figure 3:**
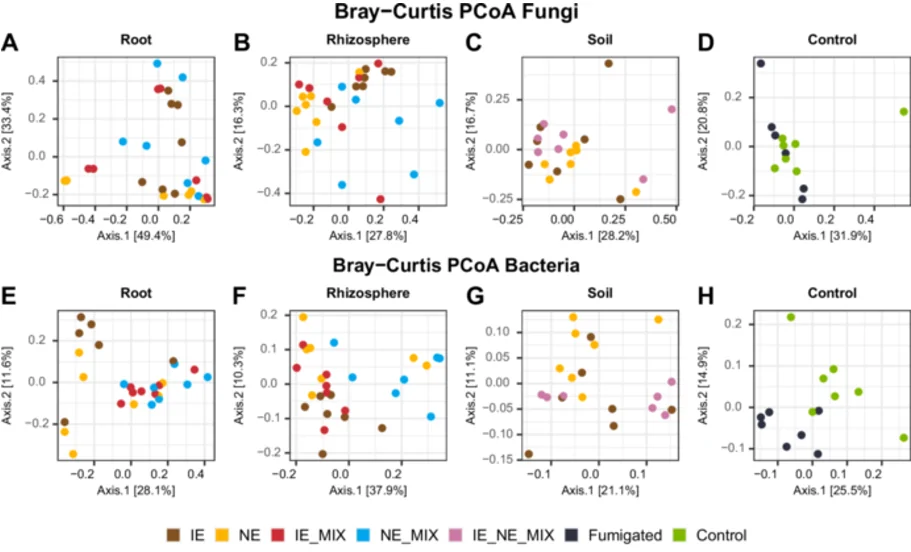
Principal Coordinates Analysis (PCoA) based on Bray‐Curtis distance showing variation in fungal (A–D) and bacterial (E–H) community composition across the different niches: root, rhizosphere, bulk soils (soil), and soils without plants (control). [Color figure can be viewed at wileyonlinelibrary.com]

**Table 1:** Effect of treatment on beta‐diversity of fungi and bacteria in root (IE, IE_MIX, NE, NE_MIX), rhizosphere (IE, IE_MIX, NE, NE_MIX), soil (IE, IE_NE_MIX, NE), and control soils (control, fumigated), as explained by Permutational Multivariate Analysis of Variance (PERMANOVA) using Bray‐Curtis distance. Multivariate homogeneity of groups DISPERSION (variances) between Treatment groups are also reported. Significant *p*‐values at 0.05 are highlighted in bold and support significant differences between Treatment groups and heterogeneity of variances between groups, permutations = 999.

PERMANOVA							DISPERSION		
Kingdom	Dataset	Df	Sum Sq	*R* ^2^	*F*‐value	*p*‐value	Sum Sq	*F*‐value	*p*‐value
Fungi	Root	3	0.701	0.161	1.541	0.148	0.077	1.117	0.360
Fungi	Rhizosphere	3	0.992	0.223	2.294	**0.001**	0.056	1.556	0.232
Fungi	Soil	2	0.300	0.111	1.126	0.296	0.003	0.088	0.903
Fungi	Control	1	0.089	0.076	0.908	0.596	0.001	0.056	0.838
Bacteria	Root	3	1.159	0.235	2.359	**0.001**	0.068	3.643	**0.022**
Bacteria	Rhizosphere	3	0.597	0.271	2.969	**0.001**	0.009	0.833	0.478
Bacteria	Soil	2	0.110	0.123	1.261	0.101	0.003	1.465	0.247
Bacteria	Control	1	0.113	0.173	2.509	**0.003**	0.002	1.174	0.323

Pairwise PERMANOVA revealed niche‐ and kingdom‐specific differences in community composition across treatment levels (Table 2). For fungal communities in the root niche, no significant pairwise differences emerged among IE, NE, IE_MIX, or NE_MIX. In contrast, in the rhizosphere, fungal communities differed significantly (*p* ≤ 0.05) between IE and NE and between NE and NE_MIX, indicating that these specific treatment combinations drove distinct community shifts. No significant fungal differences were detected in the soil niche (IE, NE, IE_NE_MIX) or between control and fumigated soils. For bacterial communities, more pronounced differences were observed among genotypes and growing conditions. In the root niche, several treatment pairs showed significant (*p* ≤ 0.05) compositional differences in beta diversity (IE vs. IE_MIX, IE vs. NE_MIX, NE vs. NE_MIX, and IE_MIX vs. NE), indicating that mixed and non‐mixed co‐cultivation strongly influenced bacterial assemblages. In the rhizosphere, bacterial communities in IE versus NE_MIX and IE_MIX versus NE_MIX also differed significantly (*p* ≤ 0.05). By contrast, soil bacterial communities were statistically similar across IE, NE, and IE_NE MIX treatments, while in control soils, only control versus fumigated showed a significant difference (*p* ≤ 0.05). These results indicate that certain treatment combinations affect fungal and bacterial beta‐diversity in distinct ways, with stronger effects generally observed in the bacterial root niche and fungal rhizosphere niche. After *p*‐value correction for multiple comparisons, none of the compared treatment groups showed differences in dispersion, supporting the significant PERMANOVA results (Table 1).

**Table 2:** Pairwise differences in beta‐diversity across treatment levels (IE, NE, IE_MIX, NE_MIX, IE_NE_MIX, control, fumigated), in root, rhizosphere, soil, and control soils of fungi and bacteria were conducted using permutational multivariate analysis of variance (PERMANOVA) with Bray‐Curtis distance matrix, and multivariate homogeneity of groups variances (DISPERSION), permutations = 999. *R* ^2^ and Benjamini & Hochberg corrected *p*‐value for multiple comparisons are provided.

Kingdom	Niche	Group 1	Group 2	Df 1	Df 2	*R* ^2^	PERMANOVA corrected *p*‐value	DISPERSION corrected *p*‐value
Fungi	Root	IE	IE_MIX	1	12	0.058	0.598	0.165
Fungi	Root	IE	NE	1	12	0.194	0.246	0.446
Fungi	Root	IE	NE_MIX	1	12	0.067	0.598	0.165
Fungi	Root	IE_MIX	NE	1	12	0.091	0.570	0.982
Fungi	Root	IE_MIX	NE_MIX	1	12	0.044	0.669	0.982
Fungi	Root	NE	NE_MIX	1	12	0.189	0.246	0.982
Fungi	Rhizosphere	IE	IE_MIX	1	12	0.120	0.100	0.386
Fungi	Rhizosphere	IE	NE	1	12	0.250	**0.006**	0.485
Fungi	Rhizosphere	IE	NE_MIX	1	12	0.147	0.096	0.096
Fungi	Rhizosphere	IE_MIX	NE	1	12	0.099	0.139	0.556
Fungi	Rhizosphere	IE_MIX	NE_MIX	1	12	0.133	0.100	0.887
Fungi	Rhizosphere	NE	NE_MIX	1	12	0.222	**0.018**	0.386
Fungi	Soil	IE	IE_NE_MIX	1	12	0.045	0.827	0.940
Fungi	Soil	IE	NE	1	12	0.097	0.270	0.940
Fungi	Soil	IE_NE_MIX	NE	1	12	0.117	0.270	0.940
Fungi	Control	control	fumigated	1	11	0.076	0.573	0.845
Bacteria	Root	IE	IE_MIX	1	11	0.196	**0.022**	0.078
Bacteria	Root	IE	NE	1	11	0.128	0.083	0.883
Bacteria	Root	IE	NE_MIX	1	11	0.248	**0.022**	0.078
Bacteria	Root	IE_MIX	NE	1	12	0.155	**0.022**	0.126
Bacteria	Root	IE_MIX	NE_MIX	1	12	0.102	0.161	0.924
Bacteria	Root	NE	NE_MIX	1	12	0.181	**0.027**	0.078
Bacteria	Rhizosphere	IE	IE_MIX	1	12	0.107	0.081	0.941
Bacteria	Rhizosphere	IE	NE	1	12	0.136	0.075	0.584
Bacteria	Rhizosphere	IE	NE_MIX	1	12	0.280	**0.012**	0.584
Bacteria	Rhizosphere	IE_MIX	NE	1	12	0.120	0.081	0.584
Bacteria	Rhizosphere	IE_MIX	NE_MIX	1	12	0.301	**0.012**	0.584
Bacteria	Rhizosphere	NE	NE_MIX	1	12	0.202	0.075	0.827
Bacteria	Soil	IE	IE_NE MIX	1	12	0.068	0.599	0.532
Bacteria	Soil	IE	NE	1	12	0.106	0.086	0.516
Bacteria	Soil	IE_NE MIX	NE	1	12	0.113	0.086	0.273
Bacteria	Control	Control	Fumigated	1	12	0.173	**0.006**	0.330

*Note:* Significant *p*‐values at 0.05 are highlighted in bold.

### 3.5 Alpha Diversity

Comparisons between median Hill 0 (i.e., richness) and Hill 1 (i.e., exponential of Shannon diversity index) values for fungi (Figure 4A–D) and bacteria (Figure 4E,F) showed significant differences (*p* ≤ 0.05) between treatment levels (IE, NE, IE_MIX, NE_MIX, IE_NE_MIX, fumigated, control) in all niches. In particular, the root niche, significantly lower median Hill 0 was detected in IE_MIX compared to IE for the fungi (Figure 4A), while IE_MIX showed lower Hill 1 for bacteria (Figure 4E). In the rhizosphere, IE_MIX showed higher Hill 0 compared to IE for the fungi, and compared to NE for the bacteria, while NE had higher median Hill 1 scores compared to other levels and NE_MIX (Figure 4B,F). In the soil niche, IE_NE_MIX had a higher Hill 0 compared to IE for the fungi, while NE was significantly higher than other treatments for bacteria. NE median Hill 1 was also different from IE_NE_MIX for the bacteria, but no difference was detected for fungi (Figure 4C,G). Finally, in the control soil samples, only bacterial communities showed a clear separation between fumigated and non‐fumigated samples (Figure 4H), whereas fungal communities in these control soils appeared less affected.

**Figure 4:**
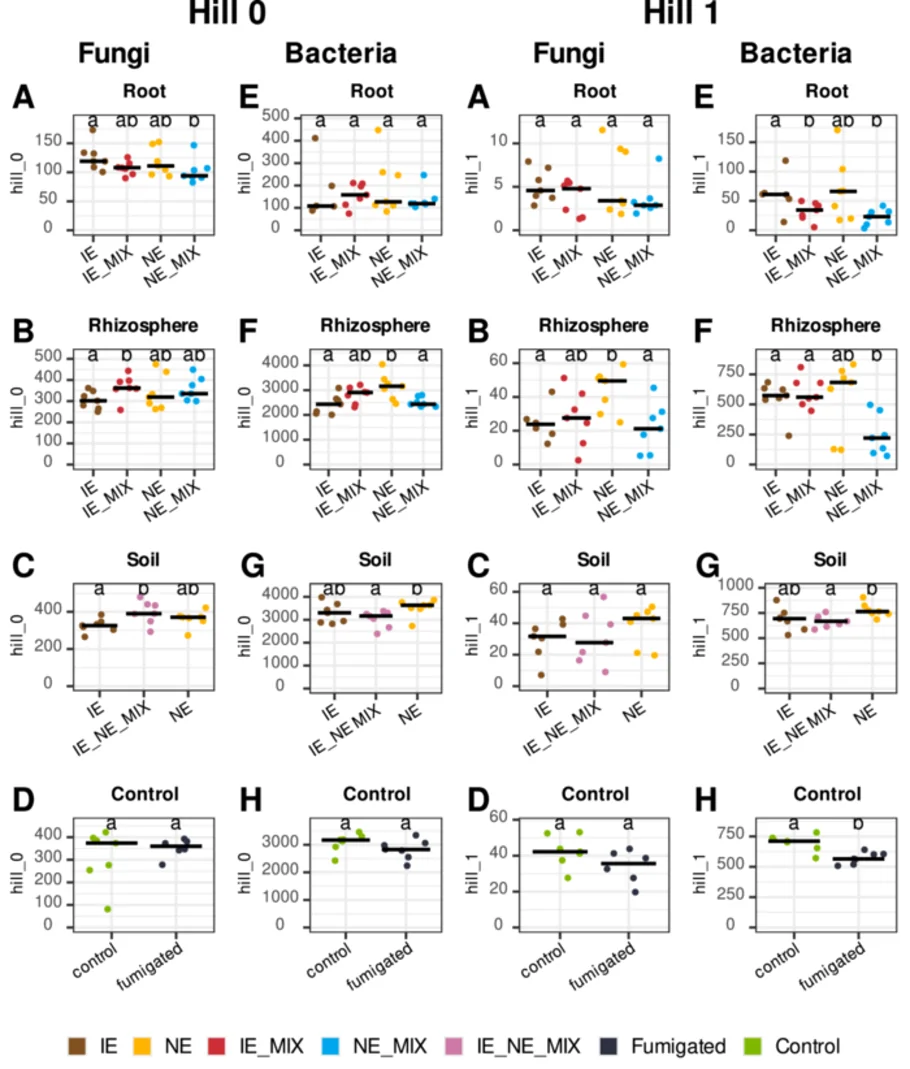
Alpha diversity measures of fungal and bacterial communities across treatments. Median Hill 0 (OTU richness; left panels) for fungi (A–D) and bacteria (E–H) and Hill 1 (Shannon‐Wiener diversity index, right panels) values for fungi (A–D) and bacteria (E–H), are represented by crossbars. Values for each sample are shown as jittered points. Different letters represent significant difference according to pairwise Wilcoxon tests (*p* ≤ 0.05) after Benjamini‐Hochberg correction. [Color figure can be viewed at wileyonlinelibrary.com]

### 3.6 Targeted Amplicon Sequencing Reveals Differential Abundance of Microbiomes in IE and NE Plants

Several taxonomic groups were detected as statistically different (*p* ≤ 0.05), in median abundance, across the different levels of treatment for both the fungi and bacteria (Figures 5, 6, 7) in different niches following the comparison highlighted by the pairwise PERMANOVA analysis (Table 2). Differentially abundant fungi were detected in the rhizosphere (Figure 5). Fungal OTUs assigned to the families of *Olpidaceae*, *Olpidium*, *Curvularia*, and *Cladophialophora humicola* were significantly more abundant in NE_MIX plants compared to NE plants. *Olpidium* was also more represented in IE than in NE plants, as well as *Leucoagaricus leucothites* and *Pyxidiophorales* (Figure 5A). Meanwhile, NE plants showed more abundance in *Stachybotryaceae*, *Sporidiobolales, Eurotiales, Trichoderma* and *Scolebasidium ramosum* compared to IE plants (Figure 5B).

**Figure 5:**
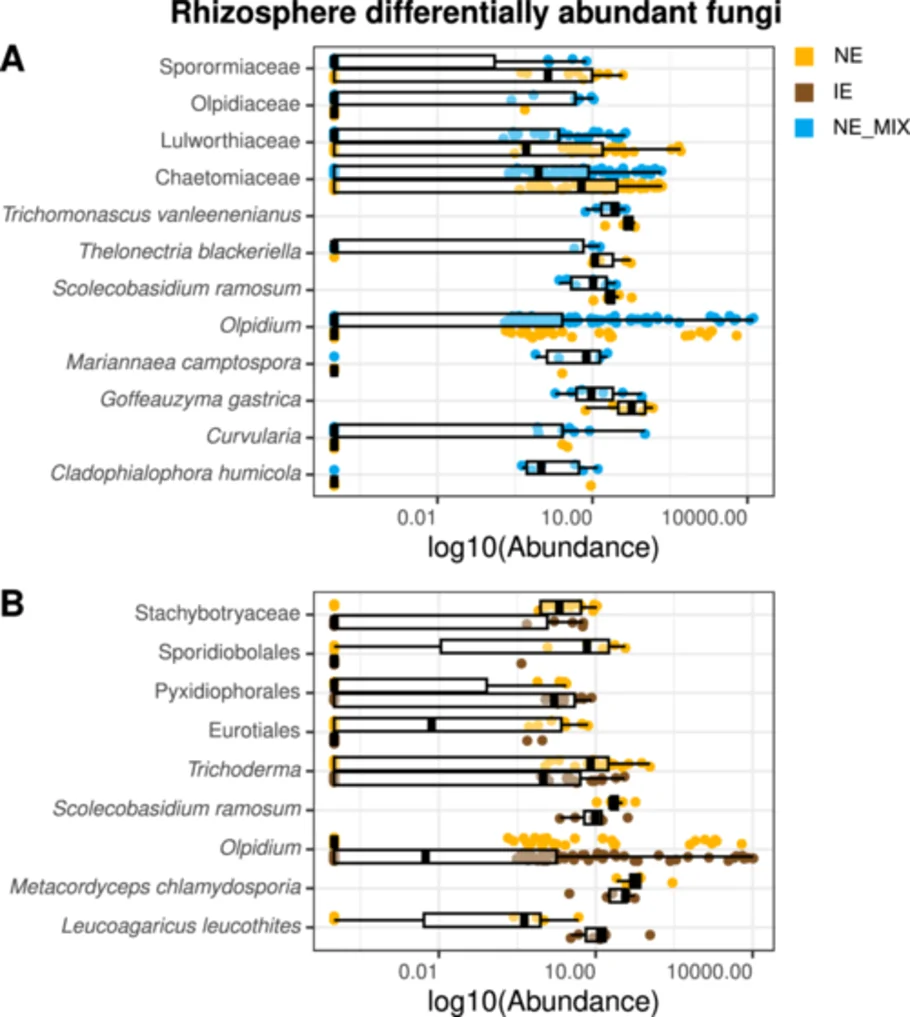
Differentially abundant fungal communities in the rhizosphere. Treatment comparisons that proved to be significant in the PERMANOVA analysis are shown: (A) NE versus NE_MIX fungal groups and (B) NE versus IE fungal groups. All groups include OTUs that were classified in that group, and each point represents an OTU in a biological sample. All groups differ significantly (*p* ≤ 0.05) between treatment levels following Wilcoxon test after Benjamini‐Hochberg *p*‐value correction. [Color figure can be viewed at wileyonlinelibrary.com]

**Figure 6:**
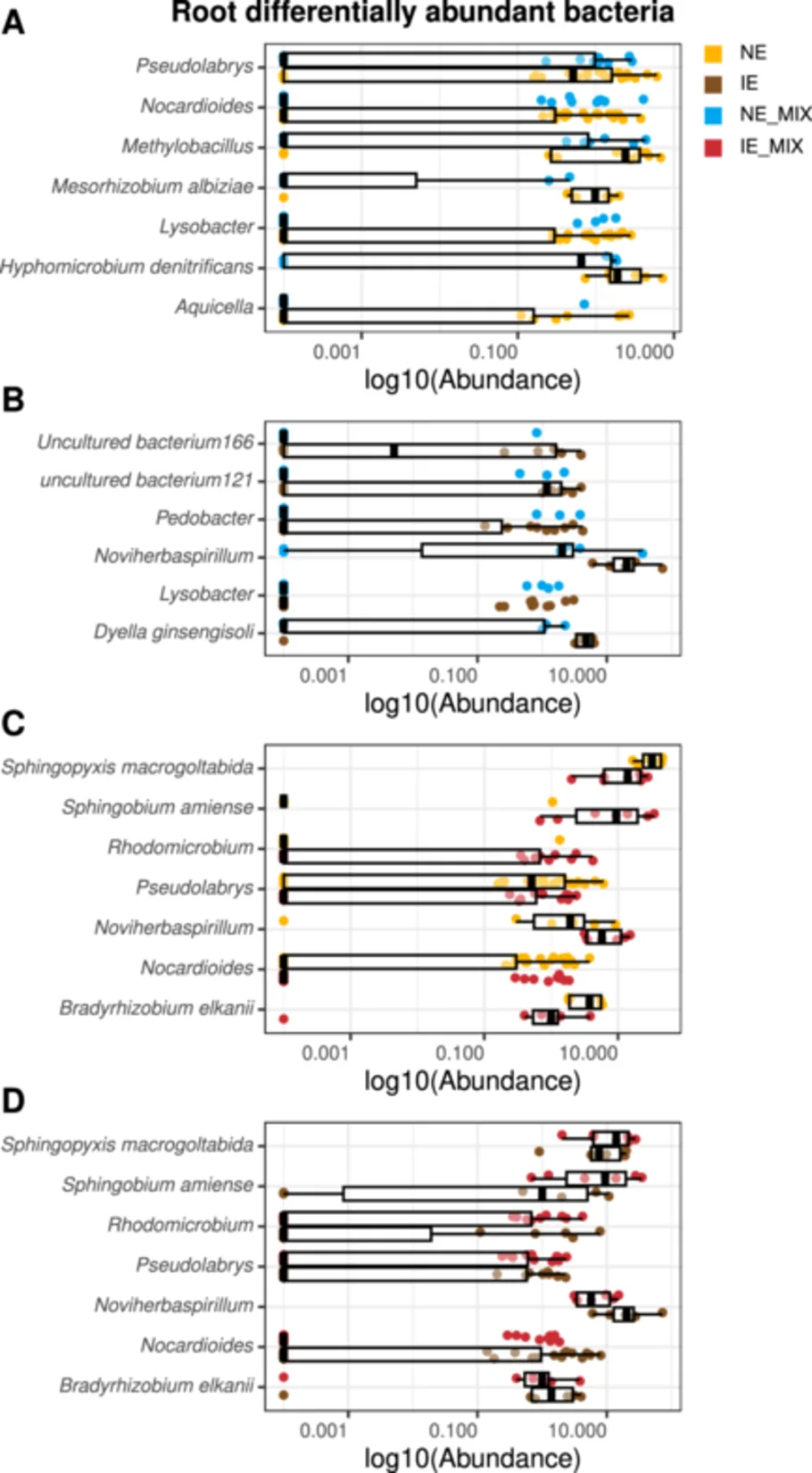
Differentially abundant bacterial communities in the roots. Treatment comparisons that showed to be significant in the PERMANOVA analysis are shown: (A) NE versus NE_MIX bacterial groups and (B) NE_MIX versus IE bacterial groups, (C) NE versus IE_MIX bacterial groups and (D) IE versus IE_MIX bacterial groups. All groups include OTUs that were classified in that group and each point represents an OTU in a biological sample. All groups significantly differ (*p* ≤ 0.05) between treatment levels using Wilcoxon test after Benjamini‐Hochberg *p*‐value correction. [Color figure can be viewed at wileyonlinelibrary.com]

**Figure 7:**
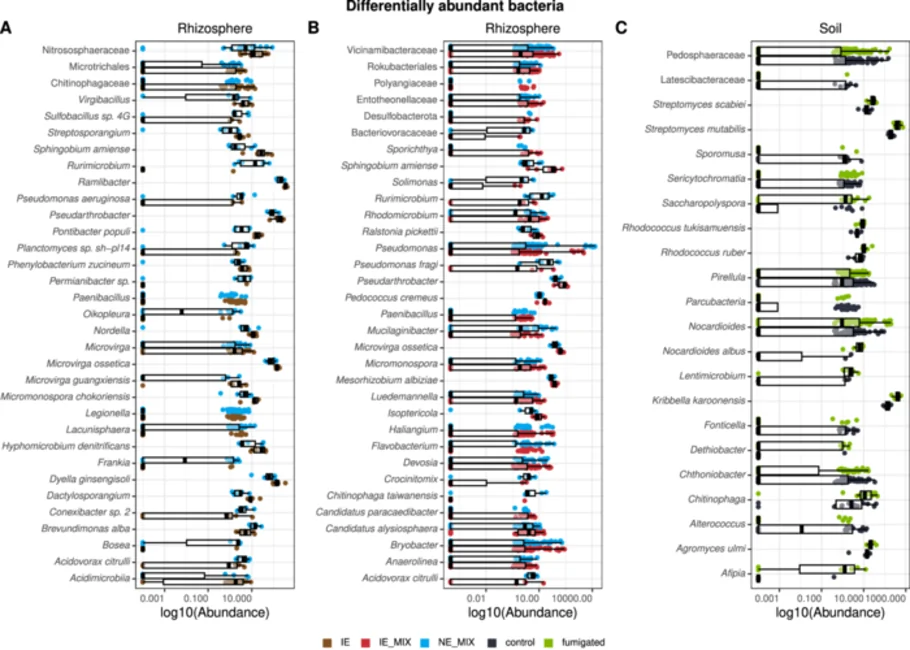
Differentially abundant bacterial communities in the rhizosphere and bulk soils. Treatment comparisons that showed to be significant in the PERMANOVA analysis are shown: (A) IE versus NE_MIX bacterial groups, (B) IE_MIX versus NE_MIX bacterial groups, and (C) isoprene fumigated versus control bacterial groups in soil. All groups include OTUs that were classified in that group and each point represents an OTU in a biological sample. All groups significantly differ (*p* ≤ 0.05) between treatment levels using Wilcoxon test after Benjamini‐Hochberg *p*‐value correction. [Color figure can be viewed at wileyonlinelibrary.com]

Regarding bacteria in the root niche (Figure 6), a limited number of taxa were found to be significantly more abundant in NE plants compared to NE_MIX plants, including *Pseudolabrys*, *Methylobacillus*, *Mesorhizobium albiziae*, *Lysobacter*, and *Hyphomicrobium denitrificans* (Figure 6A). Conversely, *Pedobacter*, *Noviherbaspirillum*, and *Dyella ginsengisoli* showed higher abundance in IE plants compared to NE_MIX plants (Figure 6B). *Bradyrhizobium elkanii*and *Pseudolabrys* were also more abundant in NE plants than in IE_MIX plants (Figure 6C). Interestingly, *Sphingobium amiense* was more abundant in IE_MIX plants compared to NE plants (Figure 6C). Additionally, *Noviherbaspirillum* was more abundant in IE_MIX plants than in NE plants (Figure 6C), as well as, more abundant in IE compared to IE_MIX (Figure 6D).

In the rhizosphere, several taxa were significantly more abundant in IE plants than in NE_MIX plants, including *Microvirga ossetica*, *Microvirga guangxiensis*, *Microvirga*, *Paenibacillus*, *Micromonospora chokoriensis*, *Dyella ginsengisoli*, and members of the *Chitinophagaceae* family (Figure 7A). The plant pathogen of cucurbits, *Acidovorax citrulli*, was more abundant in the NE_MIX plants compared to IE plants (Figure 7A) and higher in NE_MIX compared to IE_MIX (Figure 7B). Other taxonomic groups with no pathogenic properties, weakly plant‐associated evidence or plant growth promotion, such as *Crocinitomix*, *Mucilaginibacter*, *Pseudomonas fragi*, *Rurimicrobium* and *Chitinophaga taiwanensis*. Several bacterial taxa, including *Rurimicrobium*, *Paenibacillus*, *Pseudarthrobacter*, *Isoptericola*, *Mesorhizobium albiziae*, *Micromonospora*, and *Microvirga ossetica*, were also significantly more abundant in IE_MIX plants compared to NE_MIX plants (Figure 7B). No taxonomic groups that were differentially abundant in the roots or the rhizosphere were found to be present in the isoprene‐fumigated soils, aside from *Chitinophaga taiwanensis* in the rhizosphere. *Chitinophaga, Saccharopolyspora, Rhodococcus and Nocardioides* groups were more abundant in the isoprene‐fumigated soils compared to the controls (Figure 7C).

## 4 Discussion

### 4.1 Isoprene Affects Root and Shoot Development in Neighbouring Plants

The relationship between isoprene emission and plant growth is complex (Monson et al. 2021). Although isoprene confers protective advantages under extreme environmental conditions, its biosynthesis requires substantial carbon and energy resources (Sharkey et al. 2007). This allocation can lead to trade‐offs, potentially limiting growth under non‐stressful conditions (Vickers et al. 2009). In this study, we observed pronounced differences in root and shoot development between IE and NE tobacco plants. IE plants developed a more extensive root system, displaying significantly greater biomass, length, and depth compared to NE plants (Figure 1). Similarly, in Arabidopsis, IE seedlings exhibited longer primary roots at 6 days post‐germination compared with NE controls, consistent with the enhanced root growth observed in tobacco (Supporting Information S1: Figure S8). These findings are in parallel with the previous reports in poplar and Arabidopsis, where IE lines exhibited deeper primary roots and altered lateral root growth compared to NE lines (Bellucci et al. 2024; Miloradovic van Doorn et al. 2020). Such enhanced root development in IE plants may be associated with isoprene‐mediated regulation of hormonal metabolism (Frank et al. 2021), potentially contributing to improved root architecture and greater stress resilience (Bellucci et al. 2024; Dani et al. 2022). In this study we used just one tobacco transformed line, more lines would be better to guard against off target effects of the transformation. However, Miloradovic van Doorn et al. 2020 using poplar with or without RNAi knockdown of *IspS* and Bellucci et al. (2024) using Arabidopsis with and without an inserted *IspS* found similar effects on root growth and architecture. Thus, we suggest the effects we found on roots are related to the *IspS* and relative isoprene emission, as we found in transformed tobacco (Supporting Information S1: Figure S7).

NE plants displayed greater aboveground growth, with significantly higher shoot biomass, plant height, and leaf area than IE plants (Figure 2). These results corroborated earlier studies where reduced biomass was observed in IE lines (Zuo et al. 2019; Vickers et al. 2009). This pattern also confirms earlier findings in poplar—a natural isoprene‐emitter—where suppression of isoprene emission led to reduced aboveground growth. (Dani and Loreto 2022). Our results indicate that isoprene emission may exert not only direct effects on the emitting plants but also influence neighbouring individuals.

Notably, co‐cultivation of IE and NE tobacco plants led to a marked increase (20%–30%) in shoot biomass, plant height, and leaf area in both genotypes compared to those grown individually. Similarly, when Arabidopsis IE and NE seedlings were grown together in sterile conditions, both genotypes exhibited enhanced primary root growth, with IE and neighbouring NE seedlings showing longer roots compared with plants grown individually (Supporting Information S1: Figure S8). This suggests a possible synergistic interaction, wherein isoprene released by IE plants may act as a volatile signalling molecule that enhances the growth of neighbouring NE plants (Figure 2). Such non‐autonomous effects of isoprene are consistent with emerging evidence that VOCs such as isoprene act as a mechanism for mediating inter‐plant communication across spatial distances (Bergman et al. 2025).

We argue that the observed effects were elicited by isoprene emitted in the root zone (Supporting Information S1: Figure S7) rather than in the atmosphere, where isoprene concentration would be very diluted. Isoprene emission from leaves is said to be completely light dependent. However, the MEP pathway must be active in all cells to make essential isoprenoids. Even though isoprene emission in the dark is much lower than in the light, some substrate supply still persists. Miloradovic van Doorn et al. 2020 were able to demonstrate that the poplar *IspS* promoter was expressed in roots of Arabidopsis plants transformed with a GFP‐GUS construct. They also found that roots of poplar emit low levels of isoprene.

The enhanced performance of NE plants in mixed cultivation supports this signalling role and implies that isoprene emission may confer community‐level benefits in mixed plant populations. While the molecular mechanisms remain to be fully elucidated, possibilities include crosstalk with hormonal signalling, redox regulation, epigenetic priming or microbiome dynamics. These findings underscore the ecological relevance of isoprene and suggest that its role extends beyond individual plant protection to influencing plant–plant interactions and growth dynamics within a shared environment and community.

### 4.2 Isoprene Emission Shapes Microbiome

Our results demonstrate that isoprene emitted by roots (Supporting Information S1: Figure S7) exerts a selective pressure on both the fungal microbiome of the rhizosphere and bacterial microbiome of the roots. Beta diversity analyses revealed significant distinctions between IE and NE treatments, particularly in the microbiomes of the rhizosphere. Rhizosphere bacteria and fungi utilise root‐emitted volatiles as signals to locate the nutrient‐rich root zone (Schulz‐Bohm et al. 2018). In this study, rhizosphere fungal communities differed between IE and NE, suggesting that isoprene emission alters fungal community composition in the vicinity of the root. This effect was further emphasised by significant differences between NE and NE_MIX treatments, indicating that when NE plants are co‐cultivated with IE neighbours, their fungal communities are reshaped, most likely through indirect exposure to isoprene. The enrichment of *Olpidium* in both NE_MIX and IE plants indicates a positive response to isoprene exposure and raises questions as to whether isoprene is being consumed directly by *Olpidium*. This observation aligns with the broader understanding that plant‐emitted volatiles, including isoprenoids, act as semiochemicals influencing microbial colonisation patterns and behaviour (Delory et al. 2016). VOCs from roots are increasingly recognised as key mediators of belowground multitrophic interactions (Junker and Tholl 2013).

In contrast to fungi, bacterial communities exhibited stronger shifts in the root niche. Differential abundance analyses showed that bacterial communities in IE roots were compositionally distinct from those in NE roots, as well as from IE_MIX and NE_MIX roots. We found an increased abundance of *Microvirga*, *Paenibacillus*, *Micromonospora*, *Dyella*, and *Chitinophagaceae* in IE plants. Several of these are classified as bacterial plant growth‐promoting organisms (BPGP), which may indicate a selective recruitment or stimulation by isoprene, potentially contributing to improved soil microbial interactions and plant health under IE conditions. Previous studies have reported the beneficial roles of *Dyella* (Anandham et al. 2008; Dethier et al. 2025), *Paenibacillus* (Weselowski et al. 2016), *Micromonospora* (Trujillo et al. 2015) and *Microvirga* (Maghboli Balasjin et al. 2022) in promoting plant growth. The higher abundance of *Rurimicrobium*, *Paenibacillus*, *Pseudarthrobacter*, *Isoptericola*, *Mesorhizobium albiziae*, *Micromonospora*, and *Microvirga ossetica* in IE_MIX plants may suggest a selective enrichment mediated by isoprene exposure, possibly reflecting the recruitment of beneficial microbial taxa in response to IE conditions. These results suggest that isoprene may serve as a selective agent favouring beneficial microbial taxa. Selection for such taxa could have functional implications for host fitness in natural and agricultural systems. In contrast, bacterial taxa such as *Pseudolabrys* and *Methylobacillus* were more prevalent in NE roots but declined in NE_MIX roots, implying that isoprene released by neighbouring IE plants has inhibited them.

### 4.3 Co‐Cultivation With IE Alters NE Microbiomes

Co‐cultivation with IE plants significantly altered the microbiome structure of NE plants. In both the root and rhizosphere niches, NE_MIX microbial communities diverged from NE plants grown individually. In the root microbiome, NE_MIX showed a significantly lower presence of taxa such as *Mesorhizobium albiziae* and *Lysobacter*, compared to NE roots. Simultaneously, NE_MIX roots shared a suite of enriched bacterial taxa with IE and IE_MIX treatments, providing evidence of microbial convergence induced by isoprene exposure.

Microbial convergence in shared environments has been observed in other contexts, such as intercropping and plant‐soil feedback studies, where chemical cues mediate microbiome assembly and resilience (Berendsen et al. 2012). Isoprene may act as one such cue, facilitating the transfer or enrichment of microbial traits across neighbouring plants. We propose that isoprene acts as a signalling molecule that promotes the recruitment of plant growth‐promoting fungi and bacteria (Figure 8A), explaining the improved growth observed in NE plants when co‐cultivated with IE neighbours. Plant growth‐promoting rhizobacteria may, in turn, induce or prime the emission of plant volatiles (Figure 8B), often involved in plant protection against pests and pathogens (Santos et al. 2014; Chiriboga M. et al. 2018), thus further improving growth capacity and performance of NE plants co‐cultivated with IE plants (Figure 8C). Fungal communities also displayed co‐cultivation effects, though these were more pronounced in the rhizosphere than in roots. Several fungal taxa, including *Olpidium* and *Cladophialophora humicola*, were significantly enriched in NE_MIX compared to NE rhizospheres. *Olpidium* was also enriched in IE rhizospheres, suggesting it may respond directly to isoprene and serve as a shared fungal responder. These findings highlight a potential indirect route through which IE plants influence their neighbours by shaping a fungal community that can colonise adjacent roots.

**Figure 8:**
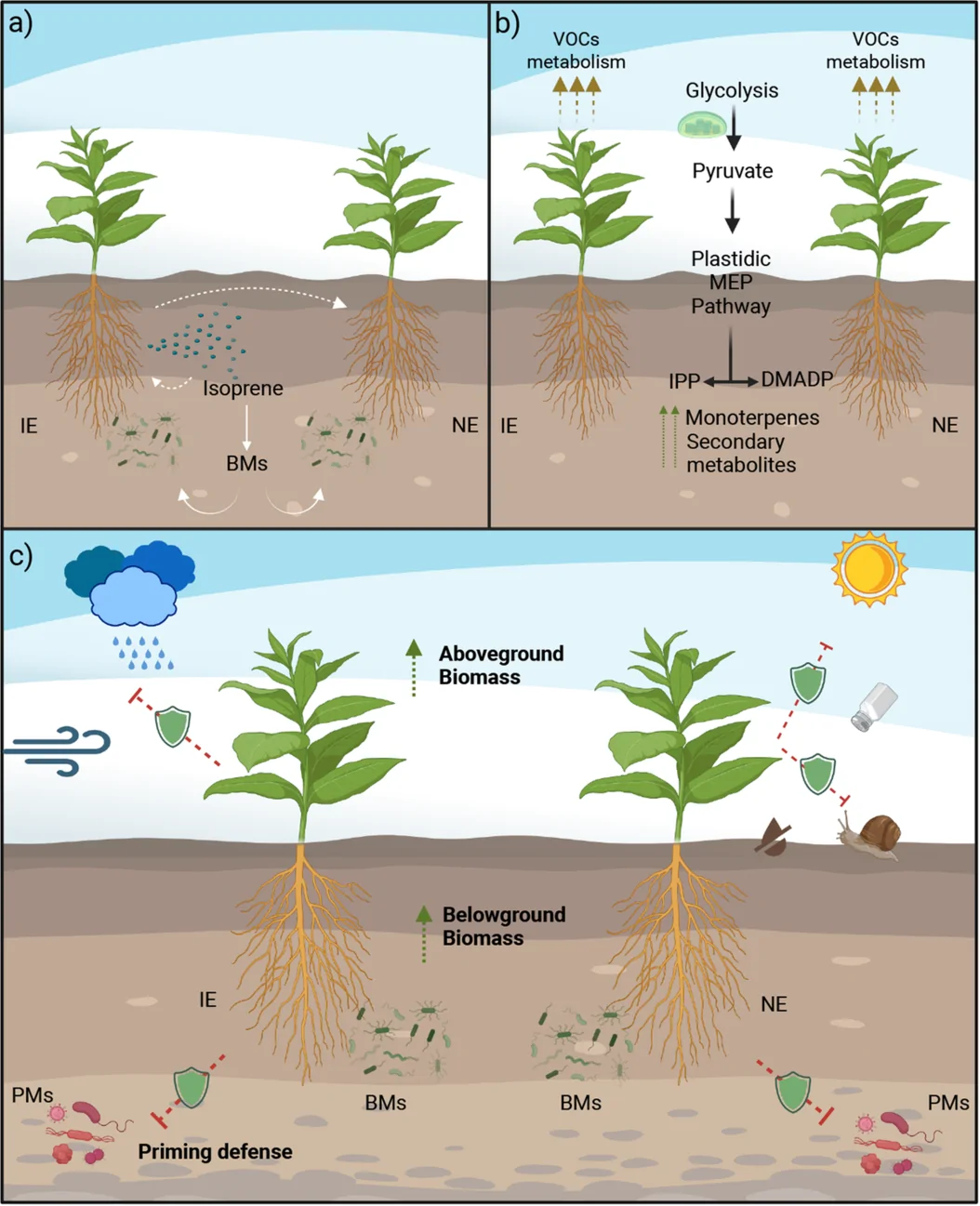
Isoprene‐emitting plants enhance ebove‐ and belowground biomass of non‐emitting plants in mixed cultivation. (A) IE plants release isoprene (Blue dot) into the soil where it interacts with beneficial microrganism. This isoprene emission attracts BMs to the root systems of both IE and NE plants. (B) BMs enhance volatile organic compound (VOC) metabolism in both IE and NE plants, including monoterpenes and other specialised compounds. (C) Isoprene increases both aboveground and belowground biomass in IE and NE plants. In addition to attracting BMs, isoprene induces a priming defence system that enhances resistance to abiotic stresses (e.g., drought, aslinity and temperature) and biotic stresses (e.g., herbivory and PMs). Abbreviation: BMs, beneficial microorganisms; IE, isoprene emitting plant; NE, non‐isoprene emitting plant; PMs, pathogenic microorganisms; green arrow indicates increasing; blue dot, isoprene. Figure was created with Biorender.com. [Color figure can be viewed at wileyonlinelibrary.com]

Isoprene fumigation of bulk soils in the absence of plants did not replicate the microbial shifts observed in living plant systems. Only a few taxa, such as *Chitinophaga taiwanensis*, appeared responsive to isoprene in soil, indicating that the plant‐microbe interface is essential for isoprene‐mediated microbiome modulation, not solely isoprene catabolism. This finding supports the hypothesis that isoprene acts within a biologically active rhizosphere context rather than as a broad‐spectrum volatile and points to the importance of BVOCs in mediating plant‐plant‐microbe communication within the rhizosphere, not only via intraspecific signalling but also shaping microbiome structure at the community level (Peñuelas et al. 2014; Schulz‐Bohm et al. 2017).

Taken together, our findings reveal that plant‐derived isoprene functions as a selective volatile cue that reshapes root‐ and rhizosphere‐associated microbial communities, structuring microbiomes that promote plant fitness in mixed communities. Previous studies indicate that isoprene functions not only as a volatile metabolite but also as a signalling molecule capable of modulating stress‐ and growth‐related gene expression and the plant metabolome (Zuo et al. 2019; Monson et al. 2021; Dani et al. 2022; Bellucci et al. 2024). These transcriptional and metabolic shifts are closely associated with hormonal reprogramming, particularly in auxin and cytokinin pathways, which are central regulators of plant growth and root architecture (Frank et al. 2021; Dani et al. 2022). At the molecular level, phosphoproteomic analyses reveal that isoprene induces rapid phosphorylation changes in proteins involved in signal transduction, redox regulation, and stress responses, supporting its role in early signalling events (Weraduwage et al. 2024). Consistently, root proteomic data demonstrate that constitutive isoprene emission reshapes protein networks linked to redox homeostasis, developmental processes, and defence (Miloradovic van Doorn et al. 2020), which are known determinants of rhizosphere interactions. These isoprene‐driven changes in plant signalling and metabolism are likely to influence the composition and function of the root‐associated microbiome by altering root exudation patterns and the physicochemical environment of the rhizosphere. In parallel, isoprene can be perceived and metabolised by soil microorganisms, leading to shifts in microbial gene expression and activity (Crombie et al. 2018; Carrión et al. 2020). Together, these findings support a model in which isoprene acts as an integrative signalling molecule linking plant physiological status with microbiome assembly and function, thereby contributing to coordinated regulation of plant growth and plant–microbe interactions.

## 5 Conclusion and Future Direction

Our findings demonstrate that plant‐emitted isoprene can influence both the phenotype and the root‐associated microbiome composition of neighbouring NE plants, and we suggest that this in turn elicits plant growth in both IE and NE plants. These results point to a potential new role for isoprene in shaping belowground ecological interactions. Notably, the strongest microbial shifts occurred in the rhizosphere and root bacterial communities, suggesting a niche‐specific microbial response to isoprene exposure. Selection for such taxa could have functional implications for host fitness in natural and agricultural systems. However, our findings are based on experiments conducted in tobacco under controlled conditions; therefore, the broader ecological relevance of these effects, including whether similar responses occur in other plant species or under natural field conditions, remains to be determined. Future research should clarify whether isoprene directly affects microbial metabolism or if the changes are caused indirectly through plant responses, for example, by using metabolomics/transcriptomics or experiments with simplified microbial communities. Additionally, exploring whether isoprene‐induced microbial changes confer functional benefits to plant health or resilience could open new perspectives for sustainable agriculture and microbiome engineering.

## Conflicts of Interest

The authors declare no conflicts of interest.

## Supporting information


**File S1:** Miseq library preparation and sequencing. **Figure S2:** Schematic representation of the sampled compartments. **Figure S3:** Distribution of (a) samples depths and (b) richness in fungi and bacteria. **Figure S4:** Rarefaction curves in fungal and bacterial microbiomes. **Figure S5:** Plots to identify best rarefaction depth for the fungal dataset. **Figure S6:** Plots to identify best rarefaction depth for the bacterial dataset. **Figure S7:** Isoprene emission from the roots of isoprene emitting (IE) and non‐emitting (NE) tobacco plants. **Figure S8:** Root phenotype in 6 days EV and IE Arabidopsis lines.

## Data Availability

The data that support the findings of this study are openly available in Sequence Read Archive at https://github.com/Gian77/Published-R-Code/tree/master/Bellucci_etal_2025_IsopreneEffectOnMicrobiome., reference number PRJNA127916. Raw fungal ITS and bacterial 16S rDNA sequence reads were deposited to the sequence read archive (Leinonen et al. 2011) under the bioproject no. PRJNA127916. All files and R code for reproducing the analyses are available at https://github.com/Gian77/Published-R-Code/tree/master/Bellucci_etal_2025_IsopreneEffectOnMicrobiome.

## References

[pce70698-bib-0001] Abarenkov, K. , R. H. Nilsson , K.‐H. Larsson , et al. 2024. “The UNITE Database for Molecular Identification and Taxonomic Communication of Fungi and Other Eukaryotes: Sequences, Taxa and Classifications Reconsidered.” Nucleic Acids Research 52: D791–D797. 10.1093/nar/gkad1039 PMC10767974 37953409

[pce70698-bib-0002] Anandham, R. , P. I. Gandhi , M. Madhaiyan , and T. Sa . 2008. “Potential Plant Growth Promoting Traits and Bioacidulation of Rock Phosphate by Thiosulfate Oxidizing Bacteria Isolated From Crop Plants.” Journal of Basic Microbiology 48: 439–447. 10.1002/jobm.200700380 18785656

[pce70698-bib-0003] Andrews, S. 2010. FastQC: A Quality Control Tool for High Throughput Sequence Data. http://www.bioinformatics.babraham.ac.uk/projects/fastqc.

[pce70698-bib-0004] Bellucci, M. , V. Locato , T. D. Sharkey , L. De Gara , and F. Loreto . 2023. “Isoprene Emission by Plants in Polluted Environments.” Journal of Plant Interactions 18: 2266463.

[pce70698-bib-0005] Bellucci, M. , M. G. Mostofa , S. M. Weraduwage , et al. 2024. “The Effect of Constitutive Root Isoprene Emission on Root Phenotype and Physiology under Control and Salt Stress Conditions.” Plant Direct 8, no. 7: e617. 10.1002/pld3.617. PMC11227114 38973810

[pce70698-bib-0006] Berendsen, R. L. , C. M. J. Pieterse , and P. A. H. M. Bakker . 2012. “The Rhizosphere Microbiome and Plant Health.” Trends in Plant Science 17: 478–486. 10.1016/j.tplants.2012.04.001 22564542

[pce70698-bib-0007] Bergman, M. E. , X.‐Q. Huang , S. Baudino , J.‐C. Caissard , and N. Dudareva . 2025. “Plant Volatile Organic Compounds: Emission and Perception in a Changing World.” Current Opinion in Plant Biology 85: 102706. 10.1016/j.pbi.2025.102706 40153896

[pce70698-bib-0008] Brilli, F. , C. Barta , A. Fortunati , M. Lerdau , F. Loreto , and M. Centritto . 2007. “Response of Isoprene Emission and Carbon Metabolism to Drought in White Poplar (*Populus Alba*) Saplings.” New Phytologist 175: 244–254. 10.1111/j.1469-8137.2007.02094.x 17587373

[pce70698-bib-0009] Caporaso, J. G. , C. L. Lauber , W. A. Walters , et al. 2011. “Global Patterns of 16S rRNA Diversity at a Depth of Millions of Sequences Per Sample.” Proceedings of the National Academy of Sciences 108: 4516–4522. 10.1073/pnas.1000080107 PMC3063599 20534432

[pce70698-bib-0010] Carrión, O. , T. J. McGenity , and J. C. Murrell . 2020. “Molecular Ecology of Isoprene‐Degrading Bacteria.” Microorganisms 8: 967. 10.3390/microorganisms8070967 PMC7409078 32605141

[pce70698-bib-0011] Chiriboga, M. X. , H. Guo , R. Campos‐Herrera , et al. 2018. “Root‐Colonizing Bacteria Enhance the Levels of (E)‐β‐caryophyllene Produced by Maize Roots in Response to Rootworm Feeding.” Oecologia 187: 459–468. 10.1007/s00442-017-4055-5 29423754

[pce70698-bib-0012] Crombie, A. T. , N. L. Larke‐Mejia , H. Emery , et al. 2018. “Poplar Phyllosphere Harbors Disparate Isoprene‐Degrading Bacteria.” Proceedings of the National Academy of Sciences 115: 13081–13086. 10.1073/pnas.1812668115 PMC6304962 30498029

[pce70698-bib-0013] Dani, K. G. S. , and F. Loreto . 2022. “Plant Volatiles as Regulators of Hormone Homeostasis.” New Phytologist 234: 804–812. 10.1111/nph.18035 35170033

[pce70698-bib-0014] Dani, K. G. S. , S. Pollastri , S. Pinosio , et al. 2022. “Isoprene Enhances Leaf Cytokinin Metabolism and Induces Early Senescence.” New Phytologist 234: 961–974. 10.1111/nph.17833 PMC9300082 34716577

[pce70698-bib-0015] Davis, N. M. , D. M. Proctor , S. P. Holmes , D. A. Relman , and B. J. Callahan . 2018. “Simple Statistical Identification and Removal of Contaminant Sequences in Marker‐Gene and Metagenomics Data.” Microbiome 6: 226. 10.1186/s40168-018-0605-2 PMC6298009 30558668

[pce70698-bib-0016] Delory, B. M. , P. Delaplace , M.‐L. Fauconnier , and P. du Jardin . 2016. “Root‐Emitted Volatile Organic Compounds: Can They Mediate Belowground Plant‐Plant Interactions?” Plant and Soil 402: 1–26.

[pce70698-bib-0017] Dethier, L. , J. R. P. Jespersen , and J. Lloyd , et al. 2025. “Isolation of a Novel Plant Growth‐Promoting *Dyella* Sp. From a Danish Natural Soil.” Environmental Microbiology Reports 17, no. 5: 634660. 10.1111/1758-2229.70186. PMC12421422 40927984

[pce70698-bib-0018] Duc, N. H. , H. T. N. Vo , C. van Doan , K. Á. Hamow , K. H. Le , and K. Posta . 2022. “Volatile Organic Compounds Shape Belowground Plant–Fungi Interactions.” Frontiers in Plant Science 13: 1046685. 10.3389/fpls.2022.1046685 PMC9763900 36561453

[pce70698-bib-0019] Edgar, R. C. 2010. “Search and Clustering Orders of Magnitude Faster Than BLAST.” Bioinformatics 26: 2460–2461. 10.1093/bioinformatics/btq461 20709691

[pce70698-bib-0020] Edgar, R. C. 2013. “UPARSE: Highly Accurate OTU Sequences From Microbial Amplicon Reads.” Nature Methods 10: 996–998. 10.1038/nmeth.2604 23955772

[pce70698-bib-0021] Edgar, R. C. , and H. Flyvbjerg . 2015. “Error Filtering, Pair Assembly and Error Correction for Next‐Generation Sequencing Reads.” Bioinformatics 31: 3476–3482. 10.1093/bioinformatics/btv401 26139637

[pce70698-bib-0022] Frank, L. , M. Wenig , A. Ghirardo , et al. 2021. “Isoprene and β‐Caryophyllene Confer Plant Resistance via Different Plant Internal Signalling Pathways.” Plant, Cell & Environment 44: 1151–1164. 10.1111/pce.14010 33522606

[pce70698-bib-0023] Gardes, M. , and T. D. Bruns . 1993. “ITS Primers With Enhanced Specificity for Basidiomycetes ‐ Application to the Identification of Mycorrhizae and Rusts.” Molecular Ecology 2: 113–118. 10.1111/j.1365-294x.1993.tb00005.x 8180733

[pce70698-bib-0024] Giordano, D. , A. Facchiano , S. D'Auria , and F. Loreto . 2021. “A Hypothesis on the Capacity of Plant Odorant‐Binding Proteins to Bind Volatile Isoprenoids Based on in Silico Evidence.” eLife 10: e66741. 10.7554/eLife.66741 PMC8221805 34161230

[pce70698-bib-0025] Guenther, A. , T. Karl , P. Harley , C. Wiedinmyer , P. I. Palmer , and C. Geron . 2006. “Estimates of Global Terrestrial Isoprene Emissions Using MEGAN (Model of Emissions of Gases and Aerosols From Nature).” Atmospheric Chemistry and Physics 6: 3181–3210.

[pce70698-bib-0027] Hill, M. O. 1973. “Diversity and Evenness: A Unifying Notation and Its Consequences.” Ecology 54: 427–432.

[pce70698-bib-0028] Junker, R. R. , and D. Tholl . 2013. “Volatile Organic Compound Mediated Interactions at the Plant‐Microbe Interface.” Journal of Chemical Ecology 39, no. 7: 810–825. 10.1007/s10886-013-0325-9 23881446

[pce70698-bib-0029] Kessler, A. , M. B. Mueller , A. Kalske , and A. Chautá . 2023. “Volatile‐Mediated Plant–Plant Communication and Higher‐Level Ecological Dynamics.” Current Biology 33: R519–R529. 10.1016/j.cub.2023.04.025 37279686

[pce70698-bib-0030] Koprivova, A. , and S. Kopriva . 2022. “Plant Secondary Metabolites Altering Root Microbiome Composition and Function.” Current Opinion in Plant Biology 67: 102227. 10.1016/j.pbi.2022.102227 35525222

[pce70698-bib-0031] Kozich, J. J. , S. L. Westcott , N. T. Baxter , S. K. Highlander , and P. D. Schloss . 2013. “Development of a Dual‐Index Sequencing Strategy and Curation Pipeline for Analyzing Amplicon Sequence Data on the MiSeq Illumina Sequencing Platform.” Applied and Environmental Microbiology 79: 5112–5120. 10.1128/AEM.01043-13 PMC3753973 23793624

[pce70698-bib-0032] Kudjordjie, E. N. , R. Sapkota , S. K. Steffensen , I. S. Fomsgaard , and M. Nicolaisen . 2019. “Maize Synthesized Benzoxazinoids Affect the Host Associated Microbiome.” Microbiome 7, no. 1: 59. 10.1186/s40168-019-0677-7 PMC6460791 30975184

[pce70698-bib-0033] Laothawornkitkul, J. , N. D. Paul , C. E. Vickers , et al. 2008. “Isoprene Emissions Influence Herbivore Feeding Decisions.” Plant, Cell & Environment 31: 1410–1415. 10.1111/j.1365-3040.2008.01849.x 18643955

[pce70698-bib-0071] Leinonen, R. , H. Sugawara , and M. Shumway . 2011. “The Sequence Read Archive.” Nucleic Acids Research 39: D19–D21. 10.1093/nar/gkq1019. PMC3013647 21062823

[pce70698-bib-0034] Liber, J. A. , G. Bonito , and G. M. N. Benucci . 2021. “CONSTAX2: Improved Taxonomic Classification of Environmental DNA Markers.” Bioinformatics 37, no. 21: 3941–3943. 10.1093/bioinformatics/btab347 33961008

[pce70698-bib-0035] Loreto, F. , and S. D'Auria . 2022. “How Do Plants Sense Volatiles Sent by Other Plants?” Trends in Plant Science 27: 29–38. 10.1016/j.tplants.2021.08.009 34544607

[pce70698-bib-0036] Loreto, F. , and S. Fineschi . 2015. “Reconciling Functions and Evolution of Isoprene Emission in Higher Plants.” New Phytologist 206: 578–582. 10.1111/nph.13242 25557381

[pce70698-bib-0037] Loreto, F. , and J. P. Schnitzler . 2010. “Abiotic Stresses and Induced BVOCs.” Trends in Plant Science 15: 154–166. 10.1016/j.tplants.2009.12.006 20133178

[pce70698-bib-0038] Maghboli Balasjin, N. , J. S. Maki , M. R. Schläppi , and C. W. Marshall . 2022. “Plant Growth‐Promoting Activity of Bacteria Isolated From Asian Rice (*Oryza Sativa* L.) Depends on Rice Genotype.” Microbiology Spectrum 10: e02787‐21. 10.1128/spectrum.02787-21 PMC9431195 35862989

[pce70698-bib-0039] Martin, M. 2011. “CUTADAPT Removes Adapter Sequences From High‐Throughput Sequencing Reads.” EMBnet.Journal 17: 10–12.

[pce70698-bib-0040] Massalha, H. , E. Korenblum , D. Tholl , and A. Aharoni . 2017. “Small Molecules Below‐Ground: The Role of Specialized Metabolites in the Rhizosphere.” Plant Journal 90: 788–807. 10.1111/tpj.13543 28333395

[pce70698-bib-0041] McMurdie, P. J. , and S. Holmes . 2013. “Phyloseq: An R Package for Reproducible Interactive Analysis and Graphics of Microbiome Census Data.” PLoS One 8: e61217. 10.1371/journal.pone.0061217 PMC3632530 23630581

[pce70698-bib-0042] Miloradovic van Doorn, M. , J. Merl‐Pham , A. Ghirardo , et al. 2020. “Root Isoprene Formation Alters Lateral Root Development.” Plant, Cell & Environment 43: 2207–2223. 10.1111/pce.13814 32495947

[pce70698-bib-0043] Monson, R. K. , S. M. Weraduwage , M. Rosenkranz , J.‐P. Schnitzler , and T. D. Sharkey . 2021. “Leaf Isoprene Emission as a Trait That Mediates the Growth‐Defense Tradeoff in the Face of Climate Stress.” Oecologia 197: 885–902. 10.1007/s00442-020-04813-7 33420520

[pce70698-bib-0045] Ninkovic, V. , M. Rensing , I. Dahlin , and D. Markovic . 2019. “Who Is My Neighbor? Volatile Cues in Plant Interactions.” Plant Signaling & Behavior 14: 1634993. 10.1080/15592324.2019.1634993 PMC6768235 31267830

[pce70698-bib-0046] Oksanen, J. , G. L. Simpson , F. G. Blanchet , et al. 2024. vegan: Community Ecology Package. R package version 2.6‐6.1. https://CRAN.R-project.org/package=vegan.

[pce70698-bib-0047] Peñuelas, J. , D. Asensio , D. Tholl , et al. 2014. “Biogenic Volatile Emissions From the Soil.” Plant Cell & Environment 37, no. 8: 1866–1891. 10.1111/pce.12340. 24689847

[pce70698-bib-0048] Quast, C. , E. Pruesse , P. Yilmaz , et al. 2012. “The SILVA Ribosomal RNA Gene Database Project: Improved Data Processing and Web‐Based Tools.” Nucleic Acids Research 41: D590–D596. 10.1093/nar/gks1219 PMC3531112 23193283

[pce70698-bib-0049] Russo, A. , S. Pollastri , M. Ruocco , M. M. Monti , and F. Loreto . 2022. “Volatile Organic Compounds in the Interaction Between Plants and Beneficial Microorganisms.” Journal of Plant Interactions 17: 840–852.

[pce70698-bib-0050] Santos, F. , M. F. G. V. Peñaflor , P. W. Paré , et al. 2014. “A Novel Interaction Between Plant‐Beneficial Rhizobacteria and Roots: Colonization Induces Corn Resistance Against the Root Herbivore *Diabrotica Speciosa* .” PLoS One 9, no. 11: e113280. 10.1371/journal.pone.0113280 PMC4236168 25405495

[pce70698-bib-0051] Schenkel, D. , M. C. Lemfack , B. Piechulla , and R. Splivallo . 2015. “A Meta‐Analysis Approach for Assessing the Diversity and Specificity of Belowground Root and Microbial Volatiles.” Frontiers in Plant Science 6: 707. 10.3389/fpls.2015.00707 PMC4568395 26442022

[pce70698-bib-0052] Schindelin, J. , I. Arganda‐Carreras , E. Frise , et al. 2012. “Fiji: An Open‐Source Platform for Biological‐Image Analysis.” Nature Methods 9: 676–682. 10.1038/nmeth.2019 PMC3855844 22743772

[pce70698-bib-0053] Schulz‐Bohm, K. , S. Gerards , M. Hundscheid , J. Melenhorst , W. de Boer , and P. Garbeva . 2018. “Calling From Distance: Attraction of Soil Bacteria by Plant Root Volatiles.” ISME Journal 12: 1252–1262. 10.1038/s41396-017-0035-3 PMC5931972 29358736

[pce70698-bib-0054] Schulz‐Bohm, K. , L. Martín‐Sánchez , and P. Garbeva . 2017. “Microbial Volatiles: Small Molecules With an Important Role in Intra‐ and Inter‐Kingdom Interactions.” Frontiers in Microbiology 8, no. 8: 2484. 10.3389/fmicb.2017.02484 PMC5733050 29312193

[pce70698-bib-0055] Seethepalli, A. , H. Guo , X. Liu , et al. 2020. “RhizoVision Crown: An Integrated Hardware and Software Platform for Root Crown Phenotyping.” Plant Phenomics 2020: 3074916. 10.34133/2020/3074916 PMC7706346 33313547

[pce70698-bib-0056] Sharifi, R. , J.‐S. Jeon , and C.‐M. Ryu . 2022. “Belowground Plant–Microbe Communications via Volatile Compounds.” Journal of Experimental Botany 73: 463–486. 10.1093/jxb/erab465 34727189

[pce70698-bib-0057] Sharifi, R. , and C. M. Ryu . 2018. “Biogenic Volatile Compounds for Plant Disease Diagnosis and Health Improvement.” Plant Pathology Journal 34, no. 6: 459–469. 10.5423/PPJ.RW.06.2018.0118 PMC6305170 30588219

[pce70698-bib-0058] Sharkey, T. D. , M. Bellucci , F. Loreto , et al. 2026. “Humboldt Review: Revisiting Plant Isoprene Emission: From Atmospheric Chemistry to Plant Stress Resilience.” Journal of Plant Physiology 318: 154712. 10.1016/j.jplph.2026.154712 41643528

[pce70698-bib-0059] Sharkey, T. D. , A. E. Wiberley , and A. R. Donohue . 2007. “Isoprene Emission From Plants: Why and How.” Annals of Botany 101: 5–18. 10.1093/aob/mcm240 PMC2701830 17921528

[pce70698-bib-0060] Simmons, T. , D. F. Caddell , S. Deng , and D. Coleman‐Derr . 2018. “Exploring the Root Microbiome: Extracting Bacterial Community Data From the Soil, Rhizosphere, and Root Endosphere.” Journal of Visualized Experiments 135: e57561. 10.3791/57561 PMC6101100 29782021

[pce70698-bib-0061] Tang, J. , G. Schurgers , and R. Rinnan . 2019. “Process Understanding of Soil BVOC Fluxes in Natural Ecosystems: A Review.” Reviews of Geophysics 57: 966–986.

[pce70698-bib-0062] Trujillo, M. E. , R. Riesco , P. Benito , and L. Carro . 2015. “Endophytic Actinobacteria and the Interaction of *Micromonospora* and Nitrogen Fixing Plants.” Frontiers in Microbiology 6: 1341. 10.3389/fmicb.2015.01341 PMC4664631 26648923

[pce70698-bib-0063] Vickers, C. E. , M. Possell , C. I. Cojocariu , et al. 2009. “Isoprene Synthesis Protects Transgenic Tobacco Plants From Oxidative Stress.” Plant, Cell & Environment 32: 520–531. 10.1111/j.1365-3040.2009.01946.x 19183288

[pce70698-bib-0064] Weraduwage, S. M. , D. Whitten , M. Kulke , A. Sahu , J. V. Vermaas , and T. D. Sharkey . 2024. “The Isoprene‐Responsive Phosphoproteome Provides New Insights Into the Putative Signalling Pathways and Novel Roles of Isoprene.” Plant, Cell & Environment 47: 1099–1117. 10.1111/pce.14776 38038355

[pce70698-bib-0065] Weselowski, B. , N. Nathoo , A. W. Eastman , J. MacDonald , and Z. C. Yuan . 2016. “Isolation, Identification and Characterization of *Paenibacillus Polymyxa* CR1 With Potentials for Biopesticide, Biofertilization, Biomass Degradation and Biofuel Production.” BMC Microbiology 16: 244. 10.1186/s12866-016-0860-y PMC5069919 27756215

[pce70698-bib-0066] White, T. , T. Bruns , S. Lee , et al. 1990. “Amplification and Direct Sequencing of Fungal Ribosomal RNA Genes for Phylogenetics.” In PCR Protocols: A Guide to Methods and Applications, 315–322. Academic Press, Inc.

[pce70698-bib-0067] Wickham, H. 2016. ggplot2: Elegant Graphics for Data Analysis. Springer‐Verlag New York.

[pce70698-bib-0069] Zuo, Z. , S. M. Weraduwage , T. Huang , and T. D. Sharkey . 2025. “How Volatile Isoprenoids Improve Plant Thermotolerance.” Trends in Plant Science 30, no. 11: 1237–1250. 10.1016/j.tplants.2025.05.004 40461293

[pce70698-bib-0070] Zuo, Z. , S. M. Weraduwage , A. T. Lantz , et al. 2019. “Isoprene Acts as a Signaling Molecule in Gene Networks Important for Stress Responses and Plant Growth.” Plant Physiology 180: 124–152. 10.1104/pp.18.01391 PMC6501071 30760638

