# Supplementary material for: Isoprene‐Emitting Transgenic Tobacco Shapes Root Microbiome and Enhances Growth of Co‐Cultivated Non‐Emitting Plants
Source: Plant Cell Environ. 2026 Jul 5;49(11):7997–8013. doi: 10.1111/pce.70698 (PMC13646678; doi:10.1111/pce.70698)
Supplement: Supplementary file 1 [file PCE-49-7997-s001.pdf]

Supplementary Materials

Isoprene-emitting transgenic tobacco shapes root microbiome and enhances growth of co-cultivated non-emitting plants

Manuel Bellucci[1,2]†, Mohammad Golam Mostofa[3*], Gian Maria Niccolò Benucci[4], Ahmad Humayan Kabir[5], Imran Khan[3], Marco Lombardi[6], Vittoria Locato[7], Gregory Bonito[4], Francesco Loreto[8], Thomas D. Sharkey[1,2,9*]

[1]Department of Energy Plant Research Laboratory, Michigan State University, East Lansing, Michigan, USA

[2]Department of Biochemistry and Molecular Biology, Michigan State University, East Lansing, MI, USA

[3]Department of Chemistry, State University of New York College of Environmental Science and Forestry, Syracuse, NY, USA

[4]Department of Plant, Soil, and Microbial Sciences, Michigan State University, East Lansing, MI, USA

[5]Department of Biology, Lamar University, Beaumont, TX, USA

[6] Department of Agroecology, Aarhus University, Tjele, Denmark

[7] Department of Science and Technology for Sustainable Development and One Health, Università Campus Bio-Medico di Roma, Rome, Italy

[8] Department of Biology, University of Naples Federico II, Naples, Italy

[9] Plant Resilience Institute, Michigan State University, East Lansing, MI, USA

†Current address: Department of Biochemistry, Purdue University, West Lafayette, IN, USA

Supplementary Files

File S1 - Miseq library preparation and sequencing.

Supplementary Figures and Tables

Fig. S2 - Schematic representation of the sampled compartments

Fig. S3 - Distribution of (a) samples depths and (b) richness in Fungi and Bacteria

Fig. S4 - Rarefaction curves in fungal and bacterial microbiomes

Fig. S5- Plots to identify best rarefaction depth for the fungal dataset

Fig. S6 - Plots to identify best rarefaction depth for the bacterial dataset

Fig. S7 Isoprene emission in roots and soil

Fig S8 Root phenotype of Arabidopsis IE and EV lines in unmixed and mixed conditions

File S1 – Supplementary text - Miseq library preparation and sequencing.

Bacterial 16S

Bacterial 16S DNA was amplified using 515f/806r (505f:GTGCCAGCMGCCGCGGTAA 806r:GGACTACHVGGGTWTCTAAT) PCR primers. PCR products were batch normalized using an Invitrogen SequalPrep DNA Normalization plate (Thermo-Fisher Scientific, Waltham, MA, USA) and product recovered from the plates pooled. The pool was concentrated and cleaned up using a QIAquick Spin column and AMPure XP magnetic beads (Beckman Coulter, USA). The pool was quality checked and quantified using a combination of Qubit dsDNA HS (Thermo Fisher Scientific, Waltham, MA, US) , Agilent 4200 TapeStation HS DNA1000 (Agilent Technology, santa Clara, CA, US) and Invitrogen Collibri Illumina Library Quantification qPCR assays (Thermo Fisher Scientific, Waltham, MA, US).

Fungal ITS

Fungal internal transcribed spacer 1 (ITS1) region was PCR-amplified using ITS1F (ITS1f:CTTGGTCATTTAGAGGAAGTAA) and ITS2 (ITS2: GCTGCGTTCTTCATCGATGC) primers and Fluidigm common oligos CS1/CS2 fused to their 5' ends. The PCR amplification was performed with the procedure of three min at 95ºC, 35 cycles of 30 s at 95ºC, 30 s at 55ºC and 1 s at 72ºC, 10 min at 72ºC using DreamTaq polymerase kit (Thermo Fisher Scientific, Waltham, MA, USA). Subsequently, the primary PCR product was amplified in a secondary PCR using dual indexed, Illumina compatible primers which target the Fluidigm CS1/CS2 oligomers at the ends of the primary PCR products. Amplicons were batch normalized using Invitrogen SequalPrep DNA Normalization plates (Thermo-Fisher Scientific, Waltham, MA, USA) and recovered product was pooled. The pool was quality checked and quantified using a combination of Qubit dsDNA HS (Thermo Fisher Scientific, Waltham, MA, US) , Agilent 4200 TapeStation HS DNA1000 (Agilent Technology, santa Clara, CA, US) and Invitrogen Collibri Illumina Library Quantification qPCR assays (Thermo Fisher Scientific, Waltham, MA, US).

Illumina sequencing

This pool was loaded onto one (1) MiSeq v2 Standard flow cell and sequencing was carried out in a 2x250bp paired end format using a MiSeq v2 500 cycle reagent cartridge. Custom sequencing and index primers complementary to the oligomers were added to appropriate wells of the reagent cartridge. After first amplification, Illumina libraries were prepared and sequenced by the RTSF Genomics Core at Michigan State University using MiSeq v2 250PE chemistry and lllumina compatible primers following the protocol described in Kozich et al. (2013). Base calling was done by Illumina Real Time Analysis (RTA) v1.18.54 and output of RTA was demultiplexed and converted to FastQ format with Illumina Bcl2fastq v2.20.0.

Supplementary Figures and Tables

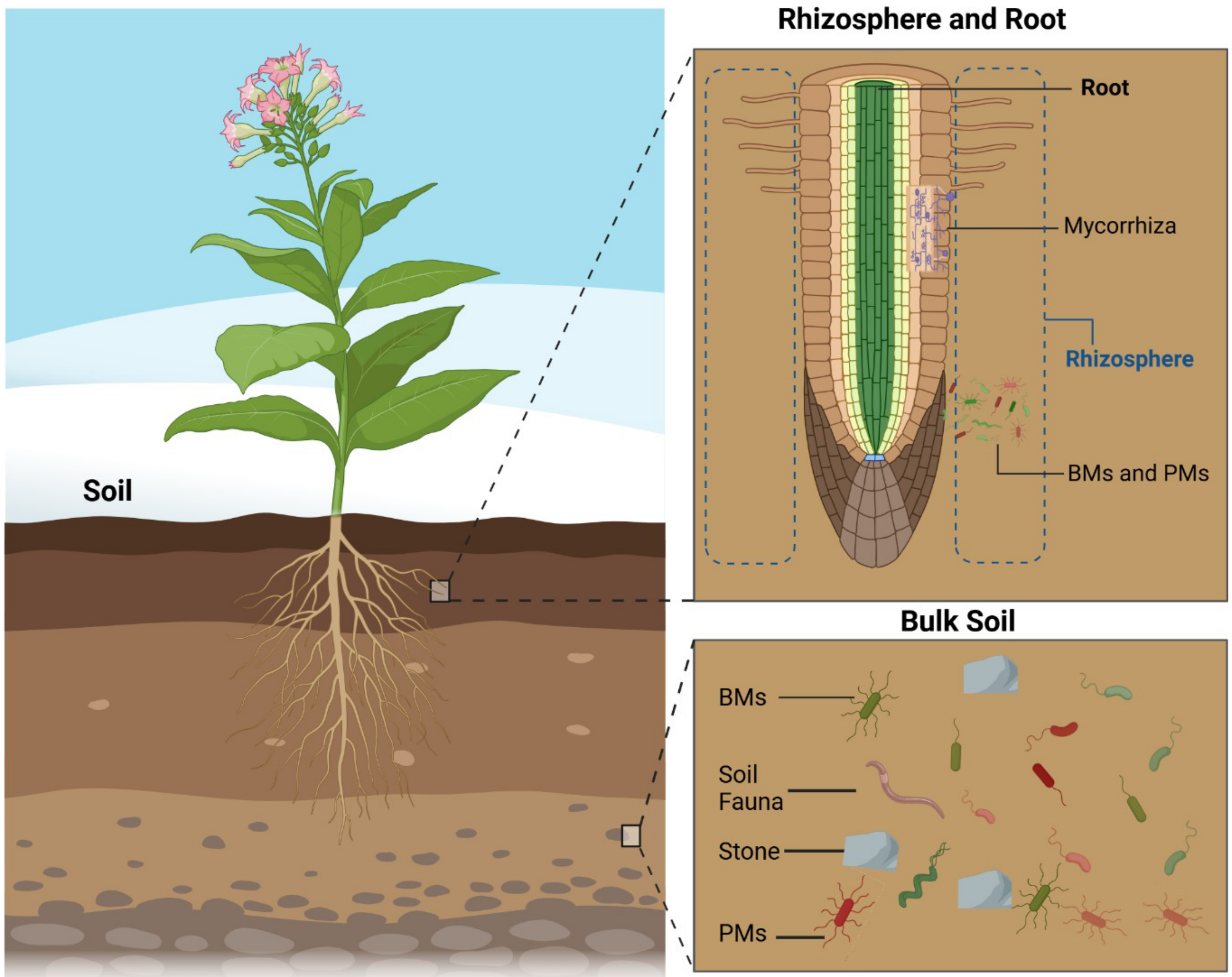

Fig. S2 - Schematic representation of the sampled compartments: bulk soil (unrooted soil distant from the root system), rhizosphere (soil tightly adhering to the root surface), and roots (washed to remove loosely attached soil). These compartments were collected to investigate plant–soil–microbe interactions.

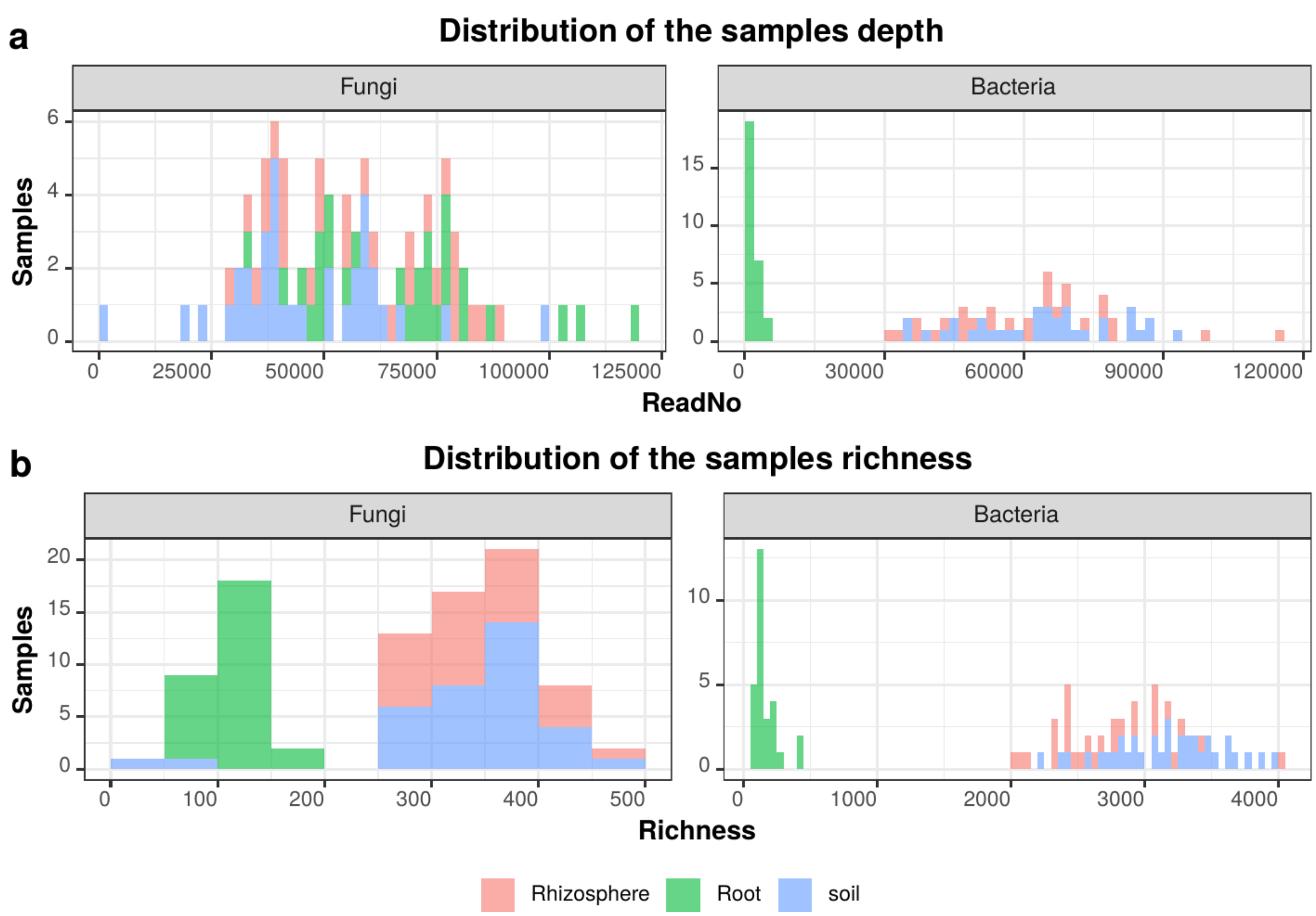

Fig. S3 - Distribution of (a) samples depths (i.e. ReadNo = number of sequence reads) and (b) richness (i.e. OTU number) in Fungi and Bacteria, respectively.

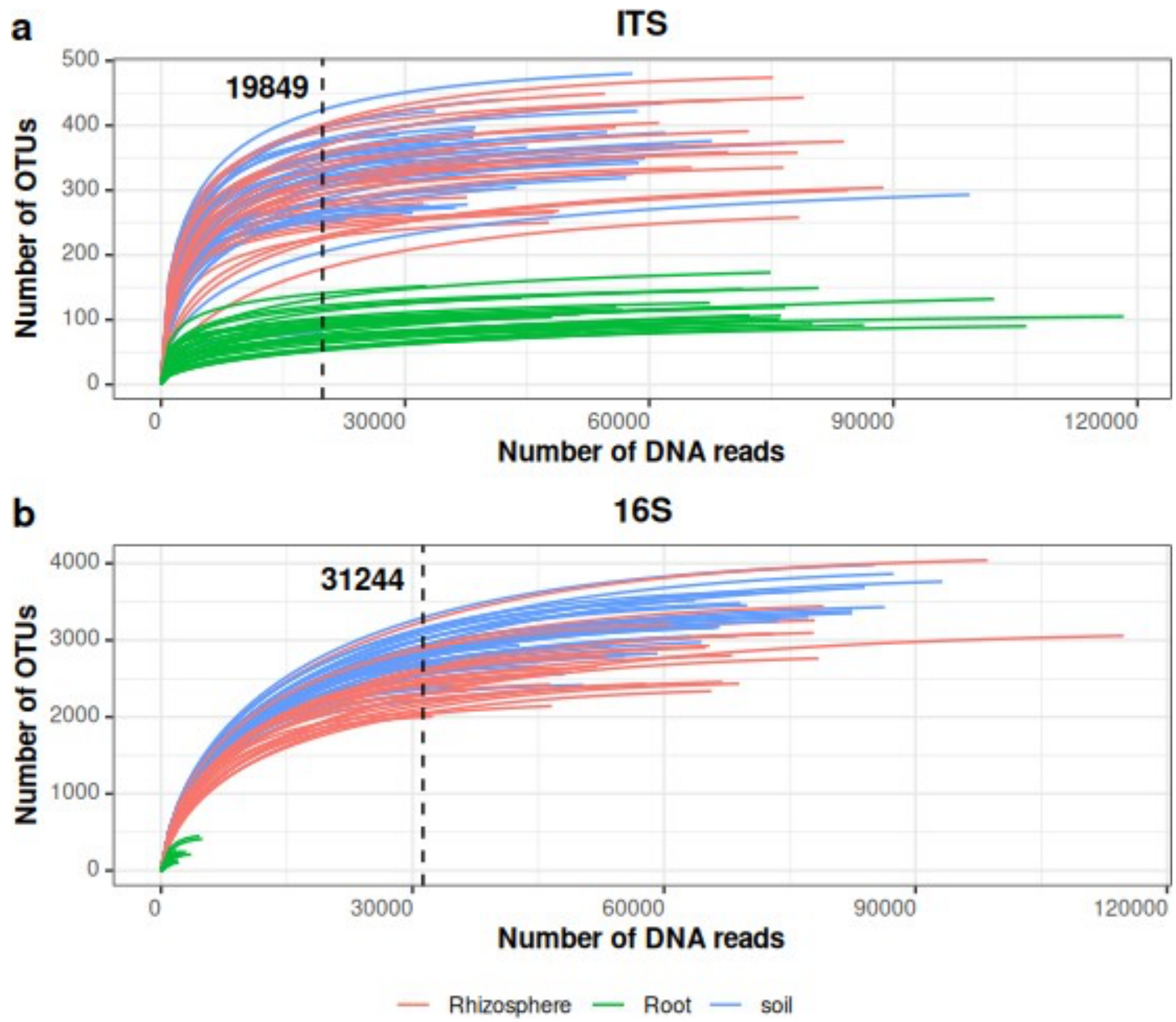

Fig. S4 - Rarefaction curves in fungal (a) and (b) bacterial microbiomes. The minimum depths for fungi (19849) and bacteria (31244) were based on soil and rhizosphere not on leaves that were rarefied separately.

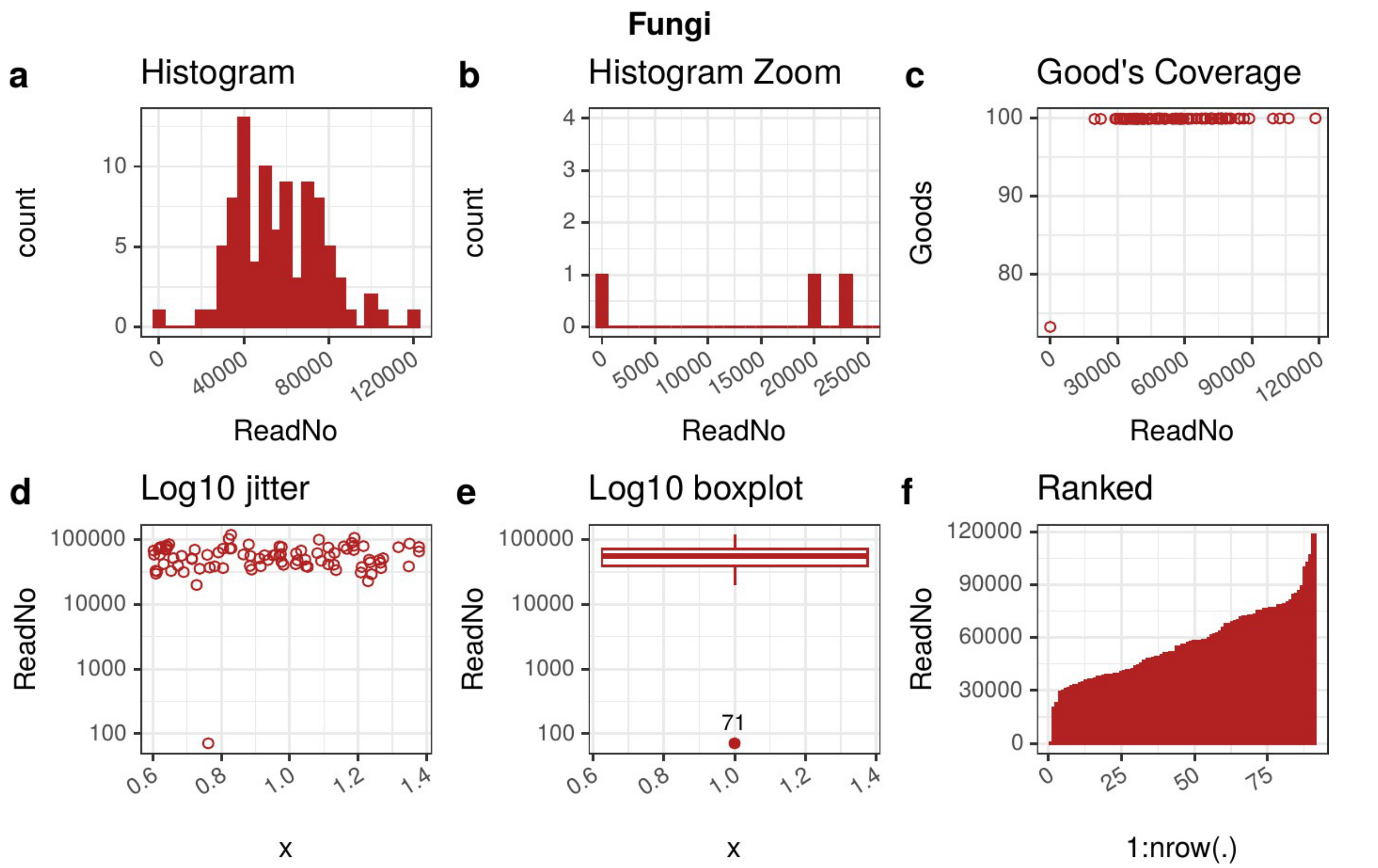

Fig. S5 - Plots to identify the best rarefaction depth for the fungal dataset. a) read number distribution, b) Zoom of sequence number distribution on the left side to the mode, c) Good's coverage for each sample, d) Jittered log10 of the sequence number for each sample, e) boxplot of the log10 or sequence number, f) samples ranked by sequence number.

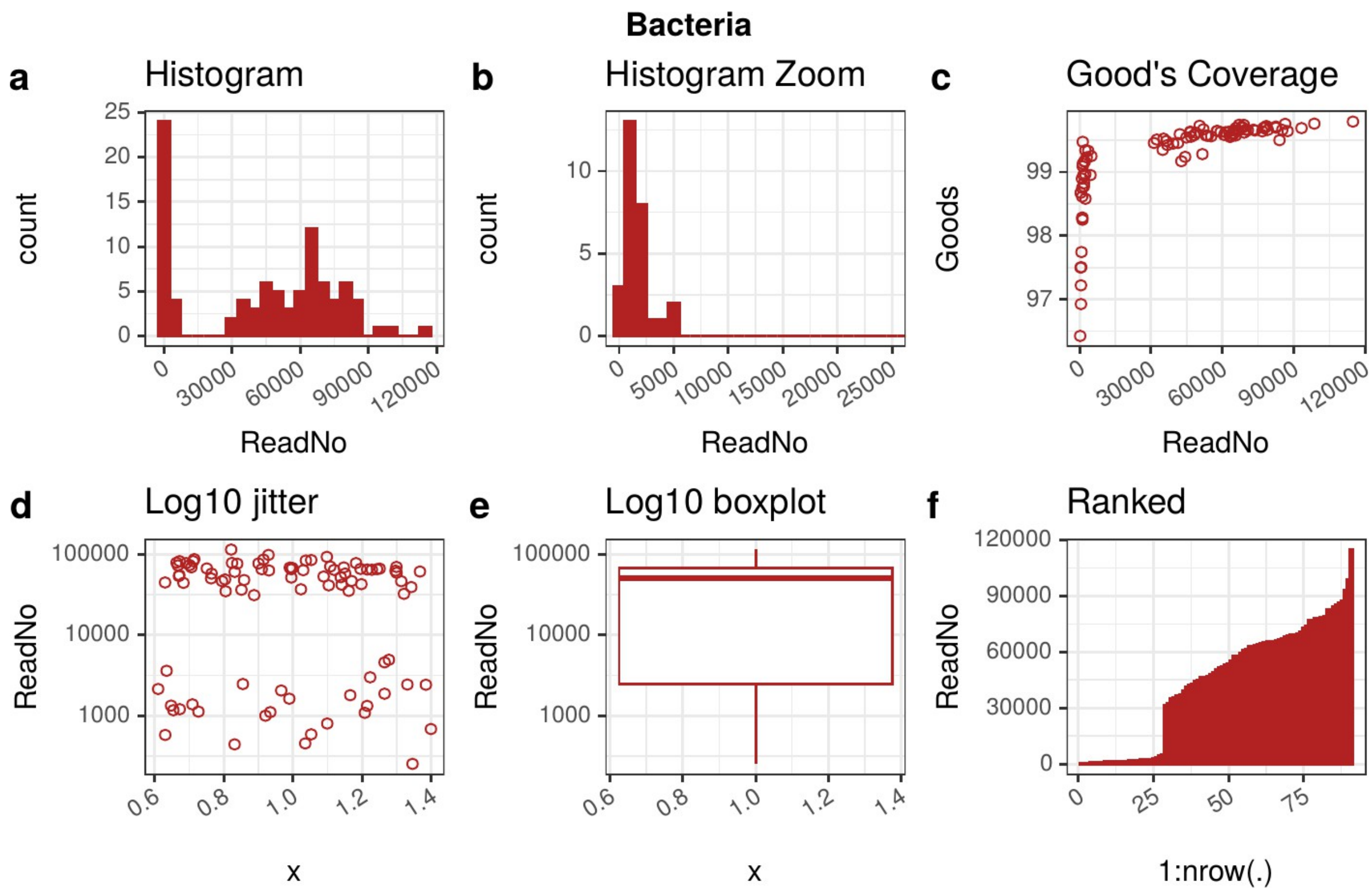

Fig. S6 - Plots to identify best rarefaction depth for the bacterial dataset. a) read number distribution, b) Zoom of sequence number distribution on the left side to the mode, c) Good's coverage for each sample, d) Jittered log10 of the sequence number for each sample, e) boxplot of the log10 or sequence number, f) samples ranked by sequence number.

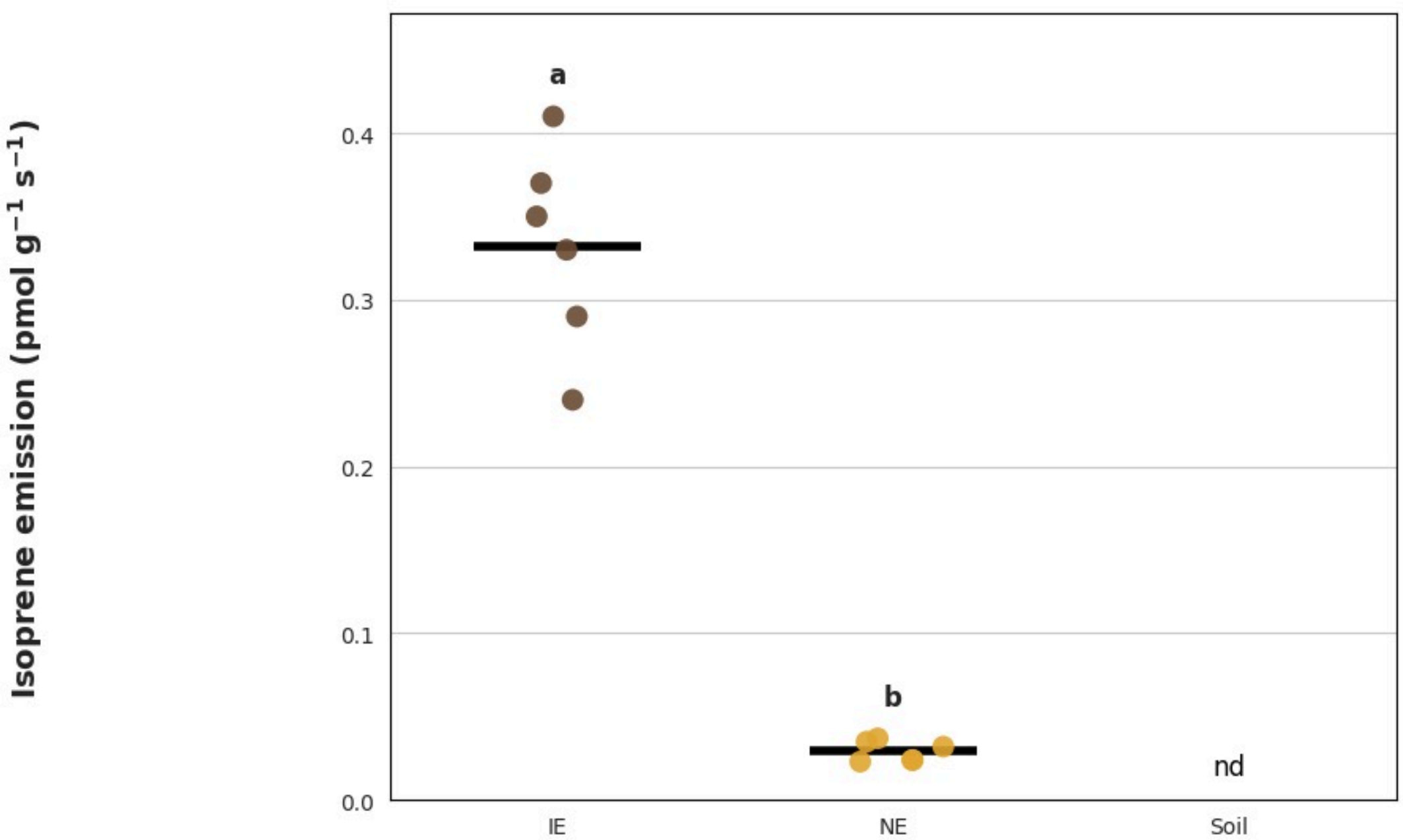

Fig S7 - Isoprene emission from the roots of isoprene emitting (IE) and non-emitting (NE) tobacco plants. Isoprene emission rates (pmol g^{-1} s^{-1}) were measured from roots of IE and NE plants and bulk soils (far from roots). Each dot represents one biological replicate (*n = 6*). The horizontal black bar indicates the mean value for each genotype. Bulk soil samples showed below the detection limit (nd). Different letters indicate statistically significant differences ($p < 0.05$) between groups of IE and NE roots.

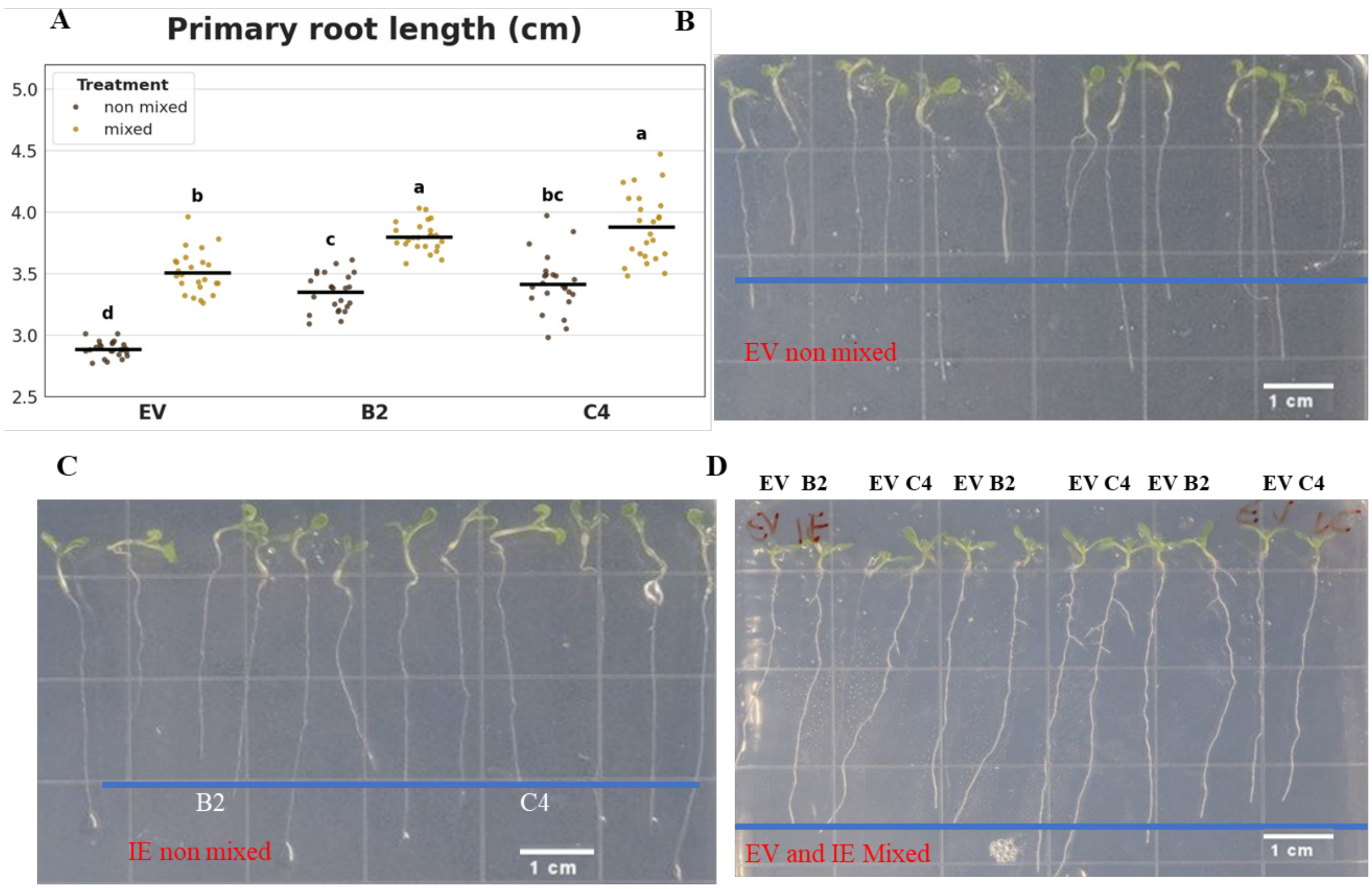

Fig S8- Root phenotype in 6 days EV and IE Arabidopsis lines. Primary root length (PRL) length of non-mixed EV (A,B) and IE (A,C) and mixed (A,D). Each dot represents one plant (n=24); horizontal bars indicate mean values. Different letters denote statistically significant differences (P < 0.05) between IE and EV plant according to one way ANOVA and post hoc Tukey test.
